# CADD-Splice—improving genome-wide variant effect prediction using deep learning-derived splice scores

**DOI:** 10.1186/s13073-021-00835-9

**Published:** 2021-02-22

**Authors:** Philipp Rentzsch, Max Schubach, Jay Shendure, Martin Kircher

**Affiliations:** 1 Charité - Universitätsmedizin Berlin, 10117 Berlin, Germany; 2 Berlin Institute of Health (BIH), 10178 Berlin, Germany; 3 Brotman Baty Institute for Precision Medicine, University of Washington, Seattle, WA 98195 USA; 4 Department of Genome Sciences, University of Washington, Seattle, WA 98195 USA

## Abstract

**Background:**

Splicing of genomic exons into mRNAs is a critical prerequisite for the accurate synthesis of human proteins. Genetic variants impacting splicing underlie a substantial proportion of genetic disease, but are challenging to identify beyond those occurring at donor and acceptor dinucleotides. To address this, various methods aim to predict variant effects on splicing. Recently, deep neural networks (DNNs) have been shown to achieve better results in predicting splice variants than other strategies.

**Methods:**

It has been unclear how best to integrate such process-specific scores into genome-wide variant effect predictors. Here, we use a recently published experimental data set to compare several machine learning methods that score variant effects on splicing. We integrate the best of those approaches into general variant effect prediction models and observe the effect on classification of known pathogenic variants.

**Results:**

We integrate two specialized splicing scores into CADD (Combined Annotation Dependent Depletion; cadd.gs.washington.edu), a widely used tool for genome-wide variant effect prediction that we previously developed to weight and integrate diverse collections of genomic annotations. With this new model, CADD-Splice, we show that inclusion of splicing DNN effect scores substantially improves predictions across multiple variant categories, without compromising overall performance.

**Conclusions:**

While splice effect scores show superior performance on splice variants, specialized predictors cannot compete with other variant scores in general variant interpretation, as the latter account for nonsense and missense effects that do not alter splicing. Although only shown here for splice scores, we believe that the applied approach will generalize to other specific molecular processes, providing a path for the further improvement of genome-wide variant effect prediction.

**Supplementary Information:**

The online version contains supplementary material available at 10.1186/s13073-021-00835-9.

## Background

One of the key steps involved in the regulation of eukaryotic gene expression is RNA splicing, the transformation of transcribed pre-mRNA into translatable mRNA through the removal of intronic sequences. While variations of this process have been described [1], the principal mechanism of RNA splicing is that the branchpoint located in the spliced intron binds to the 5′-donor site (relative to the intron), forming a lariat intermediate. The 3′-donor site binds to the acceptor and connects the two exons, thereby releasing the intron. At some genes, multiple acceptor or donor sites compete, such that multiple different alternative transcripts can be formed from one gene, i.e., alternative splicing [2]. Various studies show that more than 90% [3, 4] of genes with multiple exons undergo alternative splicing, i.e., not all exons are included in every transcript. For each exon or exon segment, the quantity “percent spliced-in” (psi) is defined as the relative fraction of transcripts this segment is included in [5]. Exons with high psi values are associated with stronger conservation and depletion of loss-of-function variation [6]. The dynamics of both canonical and alternative splicing can be influenced or disrupted by genomic sequence variation. Variants disrupting splicing are established contributors to rare genetic disease and more generally variants modulating splicing substantially contribute to phenotypic variation with respect to common traits and disease risk [7–10].

However, splicing is just one of many biological processes that can be impacted by genetic variants, with others including protein function, distal and proximal regulation of cell type-specific transcription, transcript stability, and DNA replication. Given millions of variants in a human genome [11] and myriad molecular processes through which each variant might act, pinpointing the genetic changes causal for a specific phenotype down to a set or single variant remains difficult. To address this, the field increasingly relies on automated approaches to prioritize causal variants. While some predictors specialize on certain variant categories (e.g., synonymous [12] or missense effects [13, 14]) or classes (e.g., SNVs [15] or InDels [16, 17]), others take features from different biological processes into account and enable variant interpretation across the genome. Both process-specific and genome-wide approaches to variant effect prediction have distinct advantages, and it has been challenging to reconcile them into a maximally effective approach.

A number of genome-wide scores predict variant effects from sequence alone [18, 19]; most however use annotations and genomic features defined based on experimental assays, simulations, and statistical analyses thereof [12, 20–22]. A common approach is to train machine learning classifiers to distinguish between two defined classes of variants (e.g., pathogenic and benign) using selected features. Such models can be trained via various techniques of machine learning, e.g., logistic regression, boosting trees, support vector machines, or deep learning. A general variant scoring tool that we previously developed is Combined Annotation Dependent Depletion [20, 23] (CADD), a logistic regression model that is trained on more than 15 million evolutionary derived variants (proxy-benign) and a matching set of simulated variants (proxy-deleterious). This approach has advantages over using known sets of pathogenic and benign variants. Firstly, the CADD training set is much larger, covering diverse genomic regions and even rare feature annotations. Secondly, it does not suffer from the many different ascertainment effects that come with historic and on-going selection [24] of small but well-characterized variant sets. Therefore, it leverages a high number of features and does not easily overfit.

While existing variant effect prediction scores already proved very helpful in detecting deleterious mutations genome-wide, multiple studies showed limited specificity for predicting splice-altering variants [10, 25, 26]. Even though conservation scores like PhastCons [27] or PhyloP [28], a major feature of many effect predictions, are better than random in intronic regions [26], specialized scores show improved performance and are necessary to successfully predict splice variants residing within exonic regions. There are a number of specialized scores for predicting splice changes [29], trained using different types of machine learning [30], including decision tree [31–34], probabilistic [35], and kmer-based [36, 37] models. The first generation of splicing scores, like MaxEntScan [35], focuses on the immediate neighborhood of splice junctions, as most splicing variants have been found in these regions [30]. In the last few years, more distal splicing regulatory elements have been taken into account [31, 32, 34]. Recently, deep neural networks (DNNs) achieved good results on predicting splice variants genome-wide. While the idea of using neural networks for splice predictions is more than two decades old [38], the first tool to leverage the recent progress in deep learning technology was SPANR/Spidex [39], which is trained on experimentally observed exon skipping events and predicts exon inclusion percentages based on genomic features. Instead of using predefined features, two recent tools (MMSplice [40] and SpliceAI [41]) are limited to genomic sequence as input for their prediction.

**Fig. 1:**
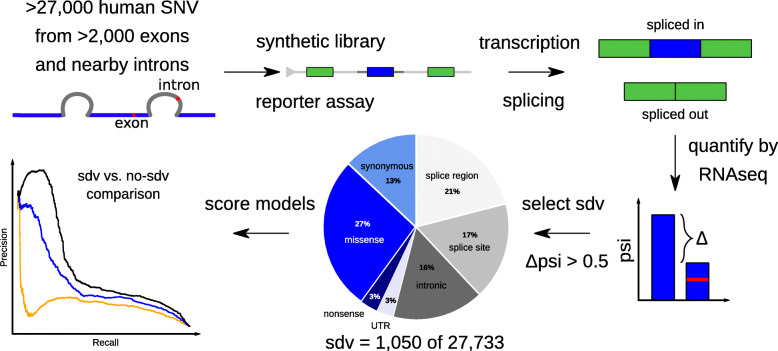
Benchmarking available splice predictions on the MFASS data set. We use the Multiplexed Functional Assay of Splicing using Sort-seq (MFASS) data set to benchmark different available splice effect predictors. MFASS studied splicing effects of more than 27,000 human exonic and intronic variants by creating a synthetic library of the respective exons (or nearest exon for intronic variants) between two GFP exons. The genome integrated sequences are transcribed and it is observed how much each exon is spliced in or out of the reporter mRNAs through RNA-seq. Changes in the percent spliced-in (psi) between reference and alternative sequence alleles are used to identify splice disrupting variants (sdv). We analyze how well different machine learning models distinguish between sdv and no-sdv variants

In order to study a large number of RNA splice-altering variants, Cheung et al. [26] developed a highly parallel reporter assay, called Multiplexed Functional Assay of Splicing using Sort-seq (MFASS, Fig. 1). The MFASS experiment used a minigene reporter assay to investigate 27,000 human population variants obtained from ExAC [42] for their impact on RNA splicing. In their analysis, the authors note that while immediate splice site variants are most important, many variants further away in the intronic and exonic sequence lead to deviation from the reference splicing behavior [26]. Due to its high number of exonic and intronic variants from over 2000 different exons tested, this data set represents a comprehensive resource for benchmarking splicing predictions. Here we present a computational analysis that leverages the MFASS data set. First, we assess several machine learning methods that score variant splicing effects. Next, we integrate the two best performing approaches into our genome-wide variant prioritization tool CADD. Finally, we show that the refined CADD model “CADD-Splice” has substantially improved performance for predicting splicing and multiple other variant categories. As process-specific information should generally improve variant prioritization, our results underline the importance of developing and integrating process-specific scores.

## Methods

### MFASS reporter assay data set of splicing effects

The MFASS [26] data set was downloaded from GitHub (https://github.com/KosuriLab/MFASS/). The data set was split into intronic (*n* = 13,603) and exonic (*n* = 14,130) variants as defined by Cheung et al. [26]. Further, the data set was split into splice-disrupting variants (sdv, *n* = 1050) and variants that do not disrupt splicing (no-sdv, *n* = 26,683) based on whether the psi ratio of the tested exon changed by more than 0.5 (Δpsi > 0.5). We explored additional thresholds at 0.7, 0.3, and 0.1, as well as using only variants with Δpsi > 0.5 for the sdv set and variants with Δpsi < 0.1 for the no-sdv set. In performance comparisons, the number of variants is slightly reduced as only variants were included for which all tested scores are defined. Psi values were downloaded in natural scale with the MFASS data set.

### Predictors of splice effects

dbscSNV v1.1 scores [33] were downloaded at https://sites.google.com/site/jpopgen/dbNSFP. The dbscSNV random forest model is shown in performance comparisons. CADD started integrating the two dbscSNV models (random forest and AdaBoost) in version 1.4. Hexamere HAL [37] scores were generated using HAL model scripts from Kipoi [43]. HAL scores including percent spliced-in (psi) were downloaded with the MFASS data set, originally obtained via the HAL website http://splicing.cs.washington.edu/ for exon skipping variants by the MFASS authors [26]. S-CAP [32] (v1.0) scores were downloaded from http://bejerano.stanford.edu/scap/. All eight S-CAP scores were combined into one score by taking the maximum per variant. Where specifically indicated and per S-CAP definition, variants without precalculated score were imputed as benign (S-CAP score = 0). Spidex [39] (v1.0, noncommercial) scores were downloaded from http://assets.deepgenomics.com/spidex_public_noncommercial_v1_0.tar.

MMSplice [40] scores were generated via the script (v1.0.2) installed from pypi. The exon-intron boundaries were provided as GTF gene annotation file downloaded from Ensembl [44] v95. The script provides model scores of the sequence with reference allele and with alternative allele for five submodels (acceptor, acceptor intron, exon, donor, and donor intron). The script also provides the composite linear models' delta_logit_psi and pathogenicity that summarize the five submodels in one metric. delta_logit_psi scores were used in performance comparisons.

Pre-scored SpliceAI [41] v1.3 scores were downloaded from Illumina BaseSpace. For larger InDels unavailable from precomputed scores, the variant scores were computed via an adapted version of the SpliceAI scripts version 1.3 (https://github.com/Illumina/SpliceAI/) that is able to integrate scores from pre-scored files in order to enable faster scoring. In comparisons of SpliceAI with other scores, all four SpliceAI models were combined into a single score by using the maximum score for a variant.

A combined score of MMSplice and SpliceAI, MMAI, was defined for evaluation on the MFASS data set. To give equal weight to both MMSplice and SpliceAI, scores were divided by their respective standard deviation across all MFASS variants (MMSplice 0.5291, SpliceAI 0.1206) and the normalized scores added. For SpliceAI, the maximum score across all SpliceAI submodels was used and for MMSplice delta_logit_psi. Similarly, MMAIpsi was defined by including normalized “percent spliced-in” as measured for the reference allele in the MFASS data set (standard deviation of 0.0622 across all MFASS variants).

We explored “proportion expressed across transcripts” (pext) [6] (version February 27, 2019) as a predictor of splice site importance. Values were downloaded from the gnomAD server and archived for reproducibility at 10.5281/zenodo.4447230. For intronic variants, the pext value of the closest exon is used.

### Integration of SpliceAI and MMSplice features in CADD

SpliceAI and MMSplice (see above) were adapted as features into CADD. For SpliceAI, all four SpliceAI submodels for 10 kb sequence windows were integrated as separate annotations. In both training data set and final scoring, predicted splice gains at annotated splice sites and predicted splice loss outside of annotated splice sites were set to 0 (for donor and acceptor sites). This was previously described for SpliceAI [41] and has been referred to as masking. We relied on precomputed SpliceAI scores as genome-wide scoring from sequence was too computationally expensive. Since models require the reference base of a variant to match the human reference, variants of the proxy-benign CADD training data set (human-derived variants) were scored with reference and alternative alleles reversed. To adjust for this, gain and loss model scores were swapped for donor and acceptor, and masking was applied after the swap as described above.

For MMSplice, all five submodels were integrated as separate annotations. MMSplice provides only scores for variants where the reference matches the genome reference. In case of the proxy-deleterious class of simulated variants as well as in scoring applications of the CADD model, the reference score was subtracted from the alternative score, as described by the authors. In the proxy-benign class, the alternative score was subtracted from the reference score. For all MMSplice submodels, positive score differences were set to 0.

For variants annotated with multiple different consequence predictions as annotated by Ensembl VEP, both MMSplice and SpliceAI scores were limited to the consequence of the same gene. All variants not annotated by MMSplice or SpliceAI were imputed as 0. All nine MMSplice annotations and SpliceAI submodels for 10 kb sequence windows were further included in a feature cross with the consequence annotation (see “Summary of CADD v1.6 models” below).

### ClinVar pathogenic vs. gnomAD common variants

ClinVar [45] was downloaded from https://ftp.ncbi.nlm.nih.gov/pub/clinvar/ (April 20, 2020). “pathogenic” variants were selected from the database based on the assignment of “Variant Clinical Significance”, excluding variants with multiple assignments. gnomAD [46] variants (version 2.1.1, 229 million single nucleotide variants from 15,708 whole genome sequenced individuals) were downloaded from https://gnomad.broadinstitute.org/. Variants were filtered based on filters set by the gnomAD authors, i.e., only variants passing quality filters were considered. InDel variants longer than 50 bp were not considered. Common variants from gnomAD with minor allele frequency (MAF) greater than 0.05 were used as a “benign set” compared to “pathogenic” ClinVar variants. In order to score GRCh37 variants with CADD GRCh38 models, variants were lifted to GRCh38 using CrossMap [47], excluding variants that did not lift back to the same GRCh37 coordinates. 12 out of 68,491 pathogenic ClinVar variants and 2300 out of 165,881 common gnomAD variants could not be lifted reciprocally between genome builds and were excluded. Variant types were annotated using Ensembl VEP [48] and CADD’s broader consequence assignments.

### ClinVar likely pathogenic vs. low frequency gnomAD variants

SNVs from ClinVar (see above) assigned clinical significance “likely-pathogenic” (incl. Variants assigned the two terms “likely-pathogenic” and “pathogenic”) were tested. We chose to also look at these variants in a separate test data set, as these are less frequently used for training of variant classifiers, reducing the likelihood of inflated performance estimates. The “likely-pathogenic” variants are compared to 300,000 randomly picked SNVs from gnomAD (see above) with minor allele frequency below 0.05 and an allele count above 1.

### Enrichment of gnomAD variants

To look at score enrichments, gnomAD variants (see above) were assigned to three bins as frequent (MAF > 0.001), rare (MAF < 0.001, allele count > 1) and singleton (allele count = 1). In order to compare between different CADD versions, score percentiles were used as variant ranks. Variant types were annotated using Ensembl VEP [48] and CADD’s broader consequence category. Enrichments per category were calculated as percentiles for all variants of the same category and dividing the number of observed variants above this threshold per bin by the number expected from random drawing. To estimate variance, 1000 bootstrap iterations were performed of which the 95% confidence interval is shown.

### Changes in CADD since version 1.4/1.5

Several minor changes compared to CADD v1.4/v1.5 were implemented as outlined in the CADD v1.6 release notes [49]. This includes annotation fixes in the GRCh38 version of CADD, specifically GERP [50] scores where an integer overflow was corrected, and Ensembl Regulatory Build [51] where the hierarchical assignment of different element categories was unstable if more than one category was reported per variant. Another issue specific to CADD v1.4/v1.5 was fixed, where highly conserved coding variants could be scored as UTR of overlapping gene annotations. Further, “unknown” was removed from the categorical consequence levels as this included only two variants in the entire training set. These variants (classified by VEP as coding sequence variants without further specification) were reassigned to the “synonymous” consequence category.

### Summary of CADD v1.6 models

A full list of annotations included in CADD-Splice is summarized in Additional file 1: Table S1 for GRCh37 and in Additional file 1: Table S2 for GRCh38. The CADD-Splice (CADD GRCh37-v1.6) model has a total of 1029 features derived from 102 annotations. Two hundred twenty-two features *X*_*i*_ derive from 90 numerical annotations and one-hot-encoding of 12 categorical/Boolean annotations. Fourteen Boolean indicators *W*_*i*_ express whether a given feature/feature group (out of cDNApos, CDSpos, protPos, aminoacid_substitution, targetScan, mirSVR, Grantham, PolyPhenVal, SIFTval, Dist2Mutation, chromHMM, dbscSNV_ada, dbscSNV_rf, and SpliceAI) is undefined. Pairs of 12 base substitutions and 189 amino acid substitutions possible to create with SNVs correspond to another 201 features. Further, 16 different variant consequence categories and a set *D* consisting of the 37 annotations bStatistic, cDNApos, CDSpos, Dst2Splice, GerpN, GerpS, mamPhCons, mamPhyloP, minDistTSE, minDistTSS, priPhCons, priPhyloP, protPos, relcDNApos, relCDSpos, relProtPos, verPhCons, verPhyloP, Dist2Mutation, freq100, freq1000, freq10000, rare100, rare1000, rare10000, sngl100, sngl1000, sngl10000, SpliceAI_accgain, SpliceAI_accloss, SpliceAI_dongain, SpliceAI_donloss, MMSplice_acceptorIntron, MMSplice_acceptor, MMSplice_donorIntron, MMSplice_donor and MMSplice_exon are used to create a set of 592 consequence interactions. The full model is fitted using the logistic regression implementation in scikit-learn is:

$$ {\beta}_0+{\sum}_{i=1}^{222}{\beta}_i{X}_i+{\sum}_{i=1}^4{\sum}_{j=1}^3{\gamma}_{ij}{\mathbbm{1}}_{\left\{\boldsymbol{i}-\boldsymbol{th}\ \boldsymbol{Ref}\ \boldsymbol{category}\ \boldsymbol{and}\ \boldsymbol{j}-\boldsymbol{th}\ \boldsymbol{Alt}\ \boldsymbol{category},\boldsymbol{i}\boldsymbol{\ne}\boldsymbol{j}\right\}}+{\sum}_{i=1}^{189}{\delta}_i{\mathbbm{1}}_{\left\{\boldsymbol{i}-\boldsymbol{th}\ \boldsymbol{amino}\ \boldsymbol{acid}\ \boldsymbol{exchange}\ \boldsymbol{possible}\ \boldsymbol{in}\ \boldsymbol{SNV}\right\}}+{\sum}_{i=1}^{14}{\tau}_i{W}_i+{\sum}_{i=1}^{16}{\sum}_{j\in D}{\alpha}_{ij}{\mathbbm{1}}_{\left\{\boldsymbol{i}-\boldsymbol{th}\ \boldsymbol{Consequence}\ \boldsymbol{category}\right\}}{X}_j $$

For CADD GRCh38-v1.6, the number of total features is 1028 derived from 120 annotations. The hyperparameter optimization strategy was unchanged from CADD v1.4 [20]. The full list of data sets used to develop CADD-Splice is provided in Additional file 1: Table S3. More information on model training (including a script for loading data matrixes and training in scikit-learn) is available at https://cadd.gs.washington.edu/training.

## Results

### Sequence-based models perform best for splice effect prediction

**Fig. 2:**
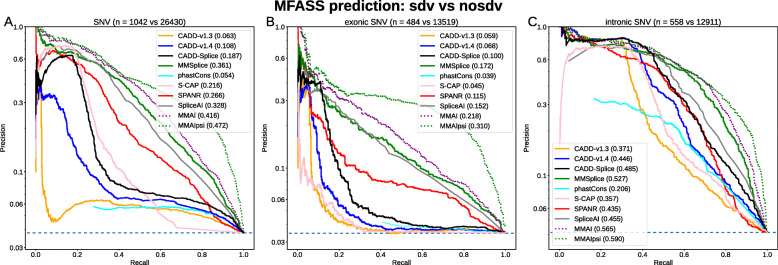
Precision-Recall performance of classifying intronic and exonic MFASS variants. Different machine learning models were used to separate splice disrupting variants from those without a splice effect. Shown are all variants in MFASS (**a**) that were scored by all splice effect predictors, **b** only exonic and **c** only intronic variants. Generally, specialized splice effect predictors, such as MMSplice, SPANR, and SpliceAI, perform better than the more general CADD, both on exonic and intronic variants. We observe the best performance by combining MMSplice and SpliceAI with the percent spliced-in (psi) value of the reference allele in a linear combination (MMAIpsi). Such a model however is assay-specific and circular with MFASS class definitions. A new CADD-Splice model, integrating MMSplice and SpliceAI as features, outperforms previous CADD models

Using the MFASS data set split into splice-disrupting variants (sdv, total *n* = 1050) and not-disrupting variants (no-sdv, *n* = 26,683, Fig. 1), we compared the performance of several recent splicing effect predictors (i.e., dbscSNV [15], HAL [37], MMSplice [40], S-CAP [32], SPANR [39], and SpliceAI [41]) and a selection of species conservation measures (Fig. 2a, Additional file 1: Fig. S1). We found that the relative performance of different scores is not dependent on the Δpsi threshold of 0.5 that was used to define sdv and no-sdv (Additional file 1: Fig. S2). We discovered that the original MFASS publication [26] inverted some scores such as PhyloP and PhastCons and that, when corrected, those scores perform better than random guessing on predicting splice effects. However, predictive power of the species conservation measures for exonic variants is limited, because most exonic variants are in the highest conservation bin (Fig. 2b). The performance of species conservation measures on intronic variants is similar to previous versions of CADD, while performance of all methods is generally better and less variable for introns (Fig. 2c). From the tested splicing effect predictors, SpliceAI and MMSplice, both DNNs based solely on genomic sequence, showed the best overall performance (Fig. 2a) with areas under the Precision Recall Curve (auPRC) of 0.328 (SpliceAI) and 0.361 (MMSplice).

Despite their similar performance on the MFASS data set, Spearman’s correlation between SpliceAI and MMSplice scores is only around 0.6. We speculate that this is due to the different model architectures. MMSplice is a convolutional neural network that was trained on data from a large massively parallel reporter assay library [37] of random sequences and takes into account 75 bp of sequence up and downstream of a known splice junction for splice donors and splice acceptors. This is in contrast to SpliceAI that, as a deep residual network, takes advantage of a much larger sequence window of 10 kbp and was trained on RNA expression data from different individuals and tissues in GTEx. We further speculated that as both scores were derived very differently, they may complement each other. Thus, we evaluated an equally weighted linear combination of the two scores (MMAI) on the MFASS data set, which indeed reached a better auPRC of 0.416 (Fig. 2).

### Percent spliced-in improves prediction only on the MFASS data set

Cheung et al. [26] showed that HAL [37] achieves the best performance on exons (auPRC 0.274, Additional file 1: Fig. S1B). However, the hexamer sequence-based model of HAL also uses psi of the reference allele as an additional assay-derived source of information. Unfortunately, the derived measure Δpsi between reference and alternative allele was used to separate sdv and no-sdv variants. psi of the reference alone separates sdv from no-sdv variants (Additional file 1: Fig. S1B, auPRC of psi 0.143, HAL with psi 0.274, HAL without psi 0.175) and interpretation of the increased performance needs to consider the underlying circularity. Adding psi in the linear combination of MMSplice and SpliceAI (MMAIpsi) gives an auPRC of 0.472 (Fig. 2a). This combination outperforms all other models on exons and much better precision is especially achieved for high recall thresholds (Fig. 2b). Using HAL without psi does result in the same performance as MMSplice (auPRC 0.175, Additional file 1: Fig. S1B), but application of HAL is by design limited to exons, which is why we chose MMSplice over HAL for a combined score.

As an assay derived measure, MFASS psi values cannot be used to predict splicing effects genome-wide, which would be a prerequisite for including them as an unbiased feature in variant prediction. While measures of psi can be derived for any RNA-Seq data set [5, 52] and are predictive of specific cell-types [53], CADD would require an organismal summary of all cell types and developmental stages. While this became available after our study [54], we explored a close proxy of psi, the proportion expressed across transcripts (pext [6]) score. pext is based on RNAseq transcript assemblies and quantifies the expression of each base in an exon in relation to the whole gene. However, neither does pext separate sdv and no-sdv variants very well (Additional file 1: Fig. S1A, auPRC of 0.058 vs 0.143 for psi) nor do we find separation of splicing variants in the CADD training set based on its value. While better equivalents may be considered, we speculate that psi values as measured in MFASS are very assay dependent.

### Extending CADD’s splice model

The performance of CADD version 1.3 compared to CADD v1.4 on the MFASS data set is very different, with auPRC increasing from 0.063 (v1.3) to 0.108 (v1.4). Up to version 1.3, CADD contained only distance information of canonical splice sites within 20 bp of variants. This had changed in CADD v1.4, where, among other annotations, dbscSNV [33] features were integrated. The dbscSNV scores are two ensemble predictors of variant splice effects around canonical splice sites (− 3 to + 8 at the 5′ splice site and − 12 to + 2 at the 3′ splice site). By splitting the MFASS data set into two sets of variants (with and without dbscSNV scores available), we found that the improvement in splice effect prediction between CADD v1.3 and CADD v1.4 was entirely dependent on this addition of dbscSNV (Additional file 1: Fig. S3). The limited distance range of the dbscSNV scores further explains why intronic variants CADD v1.4 perform similarly to PhastCons scores (which like other conservation metrics are integrated into CADD).

Based on the previous results, we added MMSplice and SpliceAI submodels as features and trained a new CADD model ‘CADD-Splice’. For MMSplice, the exon-intron boundaries required were obtained from Ensembl [44] v95 transcript models. We note that genome-wide computation of large DNNs, such as SpliceAI, can be computationally very expensive and that we therefore use pre-scored files. Nevertheless, we think that keeping features up-to-date with the latest gene annotation is crucial for providing unbiased variant scores for all genomic variants. Using purely sequence-based models such as DNNs is advantageous as scores can be updated with new gene annotations or even genome builds without retraining the model.

In preparation for integrating the DNN scores into our model, we analyzed their score distributions in the two classes of the CADD training set. We found that masking SpliceAI submodels (as recommended by the authors) benefited the annotation, as unmasked scores (i.e., splicing loss outside of existing sites and splicing gain for already existing sites) did not show class specificity (Additional file 1: Fig. S4). Similarly for the MMSplice submodels, we did not observe a depletion in the human-derived variants for positive scores (Additional file 1: Fig. S5). We therefore prepared all scores accordingly before training the model. All MMSplice and SpliceAI features were learned with positive coefficients in the CADD-Splice model, which indicates that increased scores in the splice models are associated with increased deleteriousness in the combined model.

### CADD model improvements are highly specific to splicing effects

**Fig. 3:**
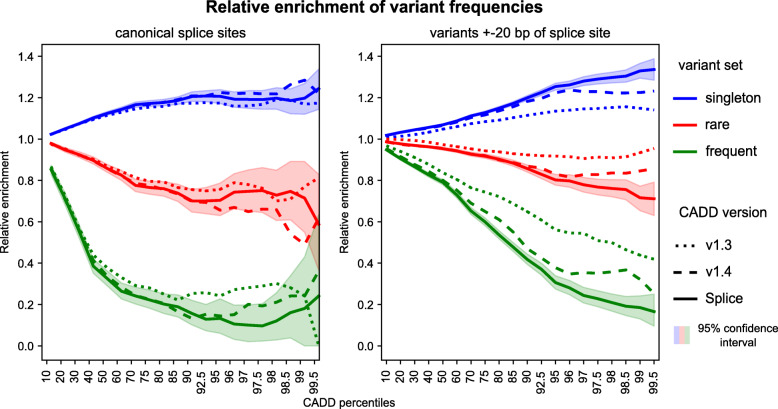
Increased enrichment for rare variants at high CADD scores. CADD assigns higher scores with increasing population frequency, despite allele frequency not being included in the model. Here, depletion and enrichment of variants is grouped by frequency and CADD score percentiles, with CADD-Splice outperforming previous versions. At high CADD scores, frequent (MAF > 0.001) and rare (allele count > 1) variants are depleted and singletons (observed once in gnomAD) enriched. For variants in canonical splice sites (left), the difference is mostly within the bootstrapped 95%-confidence interval, but CADD-Splice significantly outperforms previous versions within 20 bp of splice sites (right)

The new model, labeled CADD-Splice in all figures, shows an increased auPRC of 0.185 on the entire MFASS data set (compared to 0.108 above), with better performance on both exonic and intronic variants (Fig. 2). Still, the overall performance (across variant types) is very similar to the latest version of CADD (v1.4-GRCh37, Additional file 1: Fig. S6A) with a Spearman correlation between CADD-Splice and CADD v1.4 of 0.995 for 100,000 SNV drawn randomly from throughout the genome. Larger score changes are found for variants around known splice sites, as apparent from an increased depletion of high CADD scores for “frequent” variants (gnomAD [46] MAF > 0.1%) and an enrichment of gnomAD singletons in splice regions (Fig. 3). In the splice site proximal regions, this enrichment/depletion effect increases from CADD v1.3 over v1.4 to CADD-Splice. However, for canonical splice sites, changes are within the 95% confidence interval of the CADD-Splice measures. This can also be observed for other variant categories such as intronic variants or other coding mutations (Additional file 1: Fig. S7).

**Fig. 4:**
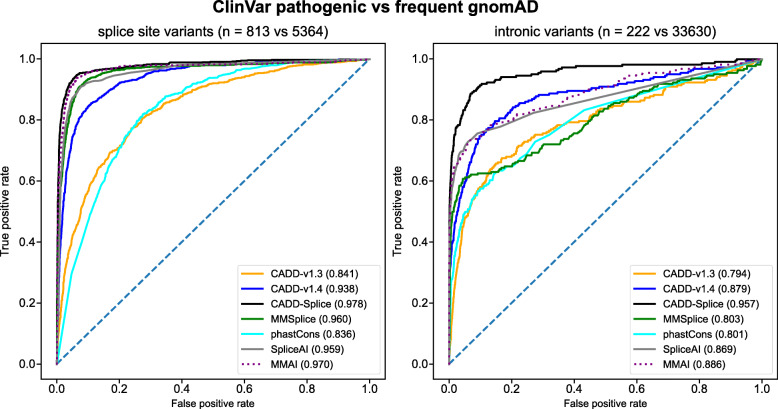
Improved performance of CADD for separating common and known pathogenic variants. The CADD-Splice model has a higher auROC than previous CADD versions and specialized splice scores in distinguishing between pathogenic variants from ClinVar and common variants (MAF > 0.05) from gnomAD for both splice site variants (left) and intronic variants (right)

In order to validate CADD-Splice on known disease-causing mutations, we used curated pathogenic variants from ClinVar and compared the area under the Receiver Operator Characteristic (auROC, Fig. 4). Rather than using curated benign variants with their respective ascertainment biases, we used common (MAF > 0.05) variants from gnomAD as controls. We observe that CADD-Splice outperforms on intronic variants (auROC 0.957) and splice site variants (auROC 0.978), not only previous versions of CADD (GRCh37-v1.4: auROC intronic 0.879 and splice site 0.938) but also the specialized scores MMSplice (0.886 and 0.970) and SpliceAI (0.869 and 0.959). For other variant categories, like synonymous and missense variants (Additional file 1: Fig. S6B-C), we observe small positive changes in model performance, probably due to a mixture of splicing-related and unrelated changes in the model.

In addition to the previous test, we compared likely-pathogenic variants from ClinVar to rare population variants (MAF < 0.05, allele count > 1) from gnomAD (Additional file 1: Fig. S8). The comparison replicates the previous results in the different variant categories, while highlighting best performance of CADD on the complete variant set. This test scenario allows comparison to the specialized splicing scores like S-CAP and SPANR whose training set partially overlaps the ClinVar pathogenic set (Additional file 1: Fig. S9). While SPANR does not perform better than CADD in any of the comparisons, S-CAP outperforms CADD on canonical splice site variants (Additional file 1: Fig. S9B) and intronic SNVs (Additional file 1: Fig. S9D). However, precomputed S-CAP scores are missing for about 9% (5980 out of 66,608) of splicing-related variants in this test set (Additional file 1: Fig. S9B-D). When interpreting missing variants as benign rather than excluding them from all comparisons (Additional file 1: Fig. S9E-H), the score’s performance reduces substantially and results only in an improved performance for canonical splice sites (Additional file 1: Fig. S9F).

Finally, we trained a CADD model using the same features and parameters as for CADD-Splice on genome build GRCh38, extending the previously described GRCh38 models [20]. In the comparison of pathogenic variants from ClinVar to common gnomAD variants, analogous to the GRCh37 model, this new CADD model (GRCh38-v1.6) scores similar to the previous model (GRCh38-v1.5) while outperforming it on splice site variants and intronic SNVs (Additional file 1: Fig. S10).

## Discussion

When analyzing genomes in research or clinical applications for phenotype causal variants, the affected molecular process is usually unknown. Therefore, genomic scores need to integrate knowledge across different processes in order to rank variants across different variant types, e.g., amino acid substitutions, truncating variants, and splicing alterations. However, to our knowledge, existing predictors scoring all types of genomic variants do not specifically take RNA splicing effects into account, as evident by their limited performance on specialized data sets [10, 25, 26]. Here we demonstrate that deep learning frameworks of splicing effects can improve the performance of existing genome-wide variant effect prediction solutions. Specifically, we show that the integration of deep learning derived scores from MMSplice and SpliceAI into the general variant effect predictor CADD enables splice effect prediction with high accuracy.

We benchmarked available splice predictions on the experimental MFASS data set and on known disease causing mutations from ClinVar. Even though MFASS does not cover some types of variants like gain-of-function mutations and deep intronic variants, it is a very valuable data set for splicing prediction and the most comprehensive data set for experimental splice-site effects today. We were able to show that existing splice models work well in predicting splice effects, provided that tools use the genomic context of each variant and not the assay-specific sequence design as input for the prediction. It further benefits methods when they are not only available as a precomputed score but provided as software that can be run genome-wide and independent of genome build and other annotations. We note that performance of all methods differs between exonic and intronic sequence (as expected due to different levels of constraints), as well as with distance to the canonical splice site. Even CADD v1.3, which uses only a 20-bp distance to canonical splice sites, has high precision in distinguishing pathogenic variants at canonical splice sites and shows reasonable performance for intronic variants. Based on the results of the benchmark sets, it is also unknown how far we can generalize observations for intronic variants that are more than 40 bp away from a known splice junction as such variants are not included in the MFASS data set and are rarely discovered from disease studies [30].

Of note, our findings contradict the original MFASS publication [26] that found HAL among the best performing predictors. We show that including psi as a feature provides an assay-specific predictive advantage and that without this feature, HAL’s performance is comparable to MMSplice and SpliceAI. While part of this observation is probably due to biases of the assay, i.e., that certain exons are more frequently integrated in the reporter construct than others, some of it could be a biological signal. More specifically, it could be argued that prevalent splice junctions (high psi) are less susceptible to disruption than less prevalent ones where multiple alternatives are generated. It has been previously observed that mutation effects scale non-monotonically with the inclusion level of an exon, with mutations having a maximum effect at a predictable intermediate inclusion level [55]. It was suggested that competition between alternative splice sites is sufficient to cause this non-linear relationship. We thought about integrating this in our model but could not determine a sensible feature. For example, the pext score, which we investigated as a genome-wide and organismal psi substitute, did not capture splice effect size.

We note that for individual cases, the joint analysis of DNA and RNA samples has proven very effective to identify and prioritize splice or regulatory variants underlying differentially expressed genes [10, 56, 57]. However, due to the tissue- and cell-type specificity of such events, informative transcriptome data is limited by the availability of the relevant RNA samples. We suggest that a combination of variant prioritization and RNA data could be very effective, and future work should explore this. For example, computational predictions could motivate the collection of relevant tissues or the establishment of cell lines from which RNA transcript data would be used to validate an actual splicing effect.

We found it very important to distinguish variants creating new splice junctions from those disrupting existing ones. SpliceAI is a prime example, as it specifically distinguishes between splice gain and loss at a particular position. Since we did not detect a depletion of predicted splice gain mutations at existing sites (and vice versa loss at non-existing sites), we were able to mask scores and to achieve a better signal to noise ratio. While MMSplice does not distinguish between gain and loss, it achieves a similar effect from integrating knowledge about the sequence of the associated donor or acceptor from the opposite site of a splice junction. This also underscores the importance of the annotation of existing splice junctions. Given that general variant classifiers such as CADD include annotations from many different sources, developers have to make sure that features are not inherently biased due to how they were generated. We are hopeful that community standards such as the upcoming Matched Annotation from NCBI and EMBL-EBI (MANE) project together with a rise of sequence-based models that can be more easily adapted to new annotations will help to produce more stable, reproducible, and better predictors.

It is clear that the significance of individual genes for specific diseases [58, 59] is not well-represented in organismal and genome-wide models of variant effects such as CADD. Existing gene and transcript specific information may therefore aid variant prioritization. For example, information about the specific phenotype (including pathways, gene interactions, or affected tissues) is potentially of high relevance. This may also motivate a more naive and inclusive approach of integrating annotations into genome-wide models. However, integrating gene and transcript-specific measures like essentiality, protein interactions and network centrality, or specificity of expression could impair the discovery of less well-studied disease genes due to observation biases [24]. To include annotations in genome-wide models, they are preferentially base-pair/substitution level resolution, available for all instances of an effect class, and do not have major biases. Thus, even though other information is useful for a final variant ranking, we are skeptical of integrating broad-scale annotations that prioritize variants based on their location in specific genomic regions.

## Conclusions

We show that process-specific DNN models are superior for identifying splice altering variants if the only possible variant effect is a splice effect. However, typically this prior knowledge is not available and variants need to be ranked across effect classes. In such a heterogeneous variant setup, a general pathogenicity predictor, like CADD, that integrates many different features, works better than the specialized splice scores in identifying pathogenic variants. The outperformance of the specialized scores is even observed when comparisons are limited to splice proximal or intronic variants. We speculate that this is due to a combination of the annotated categorical variant effects and features of species conservation. This suggests that variant prioritization can generally be improved by integrating process-specific information like splice scores. We believe that this is universal and outlines the importance of developing process-specific scores for regulatory sequences, UTRs, or non-coding RNA species.

The GRCh37 model CADD-Splice, as well as the GRCh38 model, have been released as CADD v1.6. On our website cadd.gs.washington.edu, we provide precomputed scores for all genomic SNVs, scoring of SNVs and InDels via online submission, and link to the script repository that can be used for offline scoring.

## Supplementary Information

**Additional file 1.** Supplementary Materials include Supplemental Figures (Fig. S1-S10) and Supplemental Tables (Tables S1-S3).

## Data Availability

Online variant scoring, as well as prescored files of all SNVs and selected InDels for the different versions of CADD, including CADD-Splice (released as CADD v1.6) and the used annotations are available for all noncommercial purposes at https://cadd.gs.washington.edu. Scripts for offline scoring are available at https://github.com/kircherlab/CADD-scripts [60]. The CADD v1.6 training data set is available at https://cadd.gs.washington.edu/training, including basic code for model training. All external data sets used are available under the locations specified in the Methods. Further information on the analyses is available on request.

## Abbreviations

auPRC: Area under the precision-recall curve
auROC: Area under the receiver operating characteristic
DNN: Deep neural network
MAF: Minor allele frequency
psi: Percent spliced-in
sdv: Splice disrupting variant
UTR: Untranslated region

## References

[1] Sibley CR, Blazquez L, Ule J (2016). Lessons from non-canonical splicing. Nat Rev Genet.

[2] Baralle FE, Giudice J (2017). Alternative splicing as a regulator of development and tissue identity. Nat Rev Mol Cell Biol.

[3] Wang ET, Sandberg R, Luo S (2008). Alternative isoform regulation in human tissue transcriptomes. Nature.

[4] Pan Q, Shai O, Lee LJ (2008). Deep surveying of alternative splicing complexity in the human transcriptome by high-throughput sequencing. Nat Genet.

[5] Katz Y, Wang ET, Airoldi EM, Burge CB (2010). Analysis and design of RNA sequencing experiments for identifying isoform regulation. Nat Methods.

[6] Cummings BB, Karczewski KJ, Kosmicki JA (2020). Transcript expression-aware annotation improves rare variant interpretation. Nature.

[7] Melé M, Ferreira PG, Reverter F (2015). The human transcriptome across tissues and individuals. Science.

[8] Li YI, van de Geijn B, Raj A (2016). RNA splicing is a primary link between genetic variation and disease. Science.

[9] Scotti MM, Swanson MS (2016). RNA mis-splicing in disease. Nat Rev Genet.

[10] Li X, Kim Y, Tsang EK (2017). The impact of rare variation on gene expression across tissues. Nature.

[11] Auton A, Abecasis GR, Altshuler DM (2015). A global reference for human genetic variation. Nature.

[12] Buske OJ, Manickaraj A, Mital S (2013). Identification of deleterious synonymous variants in human genomes. Bioinforma Oxf Engl.

[13] Vaser R, Adusumalli S, Leng SN (2016). SIFT missense predictions for genomes. Nat Protoc.

[14] Adzhubei I, Jordan DM, Sunyaev SR (2013) Predicting functional effect of human missense mutations using PolyPhen-2. Curr Protoc Hum Genet chapter 7:Unit7.20. doi: 10.1002/0471142905.hg0720s76. 10.1002/0471142905.hg0720s76 PMC4480630 23315928

[15] Liu X, Wu C, Li C, Boerwinkle E (2016). dbNSFP v3.0: a one-stop database of functional predictions and annotations for human nonsynonymous and splice-site SNVs. Hum Mutat.

[16] Hu J, Ng PC (2012). Predicting the effects of frameshifting indels. Genome Biol.

[17] Pagel KA, Pejaver V, Lin GN (2017). When loss-of-function is loss of function: assessing mutational signatures and impact of loss-of-function genetic variants. Bioinformatics.

[18] Zhou J, Troyanskaya OG (2015). Predicting effects of noncoding variants with deep learning-based sequence model. Nat Methods.

[19] di Iulio J, Bartha I, Wong EHM (2018). The human noncoding genome defined by genetic diversity. Nat Genet.

[20] Rentzsch P, Witten D, Cooper GM (2019). CADD: predicting the deleteriousness of variants throughout the human genome. Nucleic Acids Res.

[21] Ionita-Laza I, McCallum K, Xu B, Buxbaum JD (2016). A spectral approach integrating functional genomic annotations for coding and noncoding variants. Nat Genet.

[22] Shihab HA, Rogers MF, Gough J (2015). An integrative approach to predicting the functional effects of non-coding and coding sequence variation. Bioinforma Oxf Engl.

[23] Kircher M, Witten DM, Jain P (2014). A general framework for estimating the relative pathogenicity of human genetic variants. Nat Genet.

[24] Stoeger T, Gerlach M, Morimoto RI, Amaral LAN (2018). Large-scale investigation of the reasons why potentially important genes are ignored. Plos Biol.

[25] Mather CA, Mooney SD, Salipante SJ (2016). CADD score has limited clinical validity for the identification of pathogenic variants in noncoding regions in a hereditary cancer panel. Genet Med.

[26] Cheung R, Insigne KD, Yao D (2019). A multiplexed assay for exon recognition reveals that an unappreciated fraction of rare genetic variants cause large-effect splicing disruptions. Mol Cell.

[27] Siepel A, Bejerano G, Pedersen JS (2005). Evolutionarily conserved elements in vertebrate, insect, worm, and yeast genomes. Genome Res.

[28] Pollard KS, Hubisz MJ, Rosenbloom KR, Siepel A (2010). Detection of nonneutral substitution rates on mammalian phylogenies. Genome Res.

[29] Jian X, Boerwinkle E, Liu X (2014). In silico tools for splicing defect prediction - a survey from the viewpoint of end-users. Genet Med Off J Am Coll Med Genet.

[30] Anna A, Monika G (2018). Splicing mutations in human genetic disorders: examples, detection, and confirmation. J Appl Genet.

[31] Mort M, Sterne-Weiler T, Li B (2014). MutPred splice: machine learning-based prediction of exonic variants that disrupt splicing. Genome Biol.

[32] Jagadeesh KA, Paggi JM, Ye JS (2019). S-CAP extends pathogenicity prediction to genetic variants that affect RNA splicing. Nat Genet.

[33] Jian X, Boerwinkle E, Liu X (2014). In silico prediction of splice-altering single nucleotide variants in the human genome. Nucleic Acids Res.

[34] Soemedi R, Cygan KJ, Rhine CL (2017). Pathogenic variants that alter protein code often disrupt splicing. Nat Genet.

[35] Yeo G, Burge CB (2004). Maximum entropy modeling of short sequence motifs with applications to RNA splicing signals. J Comput Biol J Comput Mol Cell Biol.

[36] Ke S, Shang S, Kalachikov SM (2011). Quantitative evaluation of all hexamers as exonic splicing elements. Genome Res.

[37] Rosenberg AB, Patwardhan RP, Shendure J, Seelig G (2015). Learning the sequence determinants of alternative splicing from millions of random sequences. Cell.

[38] Reese MG, Eeckman FH, Kulp D, Haussler D (1997). Improved splice site detection in genie. J Comput Biol J Comput Mol Cell Biol.

[39] Xiong HY, Alipanahi B, Lee LJ (2015). RNA splicing. The human splicing code reveals new insights into the genetic determinants of disease. Science.

[40] Cheng J, Nguyen TYD, Cygan KJ (2019). MMSplice: modular modeling improves the predictions of genetic variant effects on splicing. Genome Biol.

[41] Jaganathan K, Panagiotopoulou SK, McRae JF (2019). Predicting splicing from primary sequence with deep learning. Cell.

[42] Lek M, Karczewski KJ, Minikel EV (2016). Analysis of protein-coding genetic variation in 60,706 humans. Nature.

[43] Avsec Ž, Kreuzhuber R, Israeli J (2019). The Kipoi repository accelerates community exchange and reuse of predictive models for genomics. Nat Biotechnol.

[44] Aken BL, Ayling S, Barrell D, et al. The Ensembl gene annotation system. Database. 2016, 2016:baw093. 10.1093/database/baw093. 10.1093/database/baw093 PMC4919035 27337980

[45] Landrum MJ, Lee JM, Benson M (2018). ClinVar: improving access to variant interpretations and supporting evidence. Nucleic Acids Res.

[46] Karczewski KJ, Francioli LC, Tiao G (2020). The mutational constraint spectrum quantified from variation in 141,456 humans. Nature.

[47] Zhao H, Sun Z, Wang J (2014). CrossMap: a versatile tool for coordinate conversion between genome assemblies. Bioinformatics.

[48] McLaren W, Gil L, Hunt SE (2016). The Ensembl variant effect predictor. Genome Biol.

[49] Rentzsch P, Kircher M. CADD v1.6 release notes; 2020. https://cadd.gs.washington.edu/static/ReleaseNotes_CADD_v1.6.pdf.

[50] Davydov EV, Goode DL, Sirota M (2010). Identifying a high fraction of the human genome to be under selective constraint using GERP++. Plos Comput Biol.

[51] Zerbino DR, Wilder SP, Johnson N (2015). The Ensembl regulatory build. Genome Biol.

[52] Shen S, Park JW, Huang J (2012). MATS: a Bayesian framework for flexible detection of differential alternative splicing from RNA-Seq data. Nucleic Acids Res.

[53] Park E, Pan Z, Zhang Z (2018). The expanding landscape of alternative splicing variation in human populations. Am J Hum Genet.

[54] Ling JP, Wilks C, Charles R (2020). ASCOT identifies key regulators of neuronal subtype-specific splicing. Nat Commun.

[55] Baeza-Centurion P, Miñana B, Schmiedel JM (2019). Combinatorial genetics reveals a scaling law for the effects of mutations on splicing. Cell.

[56] Anderson D, Baynam G, Blackwell JM, Lassmann T (2019). Personalised analytics for rare disease diagnostics. Nat Commun.

[57] Mohammadi P, Castel SE, Cummings BB (2019). Genetic regulatory variation in populations informs transcriptome analysis in rare disease. Science.

[58] Havrilla JM, Pedersen BS, Layer RM, Quinlan AR (2019). A map of constrained coding regions in the human genome. Nat Genet.

[59] Abramovs N, Brass A, Tassabehji M (2020). GeVIR is a continuous gene-level metric that uses variant distribution patterns to prioritize disease candidate genes. Nat Genet.

[60] Rentzsch P, Schubach M, Shendure J. Martin Kircher kircherlab/CADD-scripts: CADD version 1.6. GitHub. 2021. 10.5281/zenodo.4446709.

