# Supplementary material for: CADD-Splice—improving genome-wide variant effect prediction using deep learning-derived splice scores
Source: Genome Med. 2021 Feb 22;13:31. doi: 10.1186/s13073-021-00835-9 (PMC7901104; doi:10.1186/s13073-021-00835-9)
Supplement: Supplementary file 1 — Additional file 1. Supplementary Materials include Supplemental Figures (Fig. S1-S10) and Supplemental Tables (Tables S1-S3). [file 13073_2021_835_MOESM1_ESM.pdf]

CADD-Splice – improving genome-wide variant effect prediction using deep learning-derived splice scores

Philipp Rentzsch, Max Schubach, Jay Shendure, Martin Kircher

Additional File 1: Supplementary Materials

Supplemental Figures

Fig S1: Performance of additional scores on the MFASS data set. Precision-Recall (PR) curves of percent spliced-in (psi), pext and other scores for (A) all, (B) exonic, or (C) intronic MFASS variants. psi scores are superior to the potential genome wide replacement pext on exonic MFASS variants. For exonic variants, the hexamer score HAL is a very good splicing predictor. The HALpsi model takes psi into account and performs better than other scores. The combined SpliceAI and MMSplice score (MMAI, see also Fig. 2) can be linearly combined with psi (MMAIpsi) and outperforms HALpsi. On intronic variants (C) where pext and HAL are not defined, the auPRC of psi is smaller but also significantly different from random.

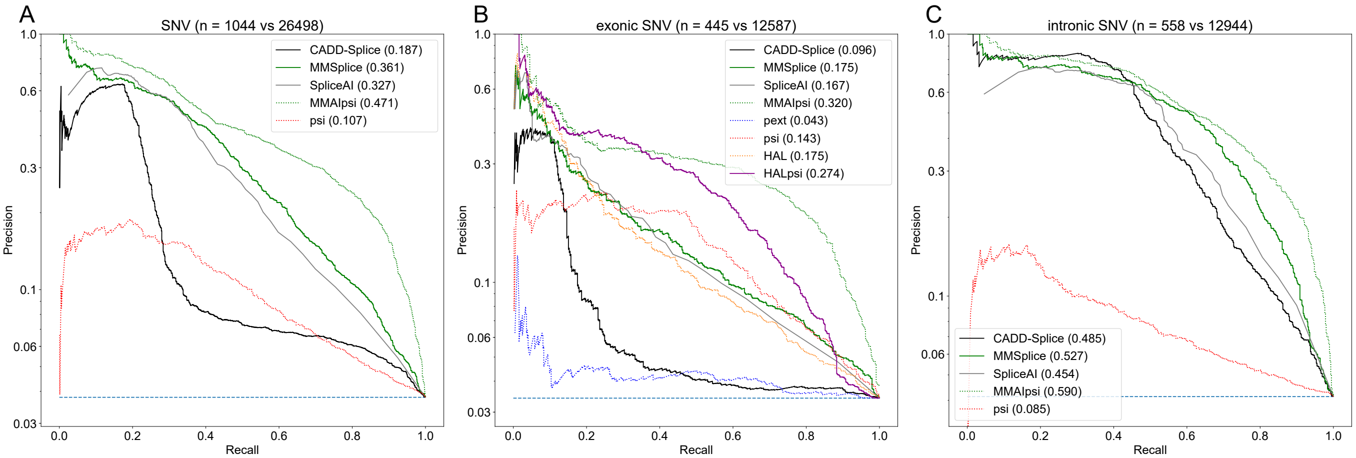

Fig S2: Using the MFASS data set with different Δpsi thresholds. The distinction of MFASS variants in splice disrupting and not-disrupting is made entirely based on the "change in psi" (Δpsi) values. The threshold of 0.5 proposed in the MFASS publication is a compromise between high effect size and a sufficiently large number of splicing disruption variants (sdv). Higher thresholds (A: Δpsi > 0.7) decrease, while lower thresholds (B: Δpsi > 0.3, C: Δpsi > 1) increase the number of sdv. While score performances change slightly depending on the threshold, the relative results are very similar. This is also true when excluding uncertain variants with Δpsi between 0.5 and 0.1 from analysis (D).

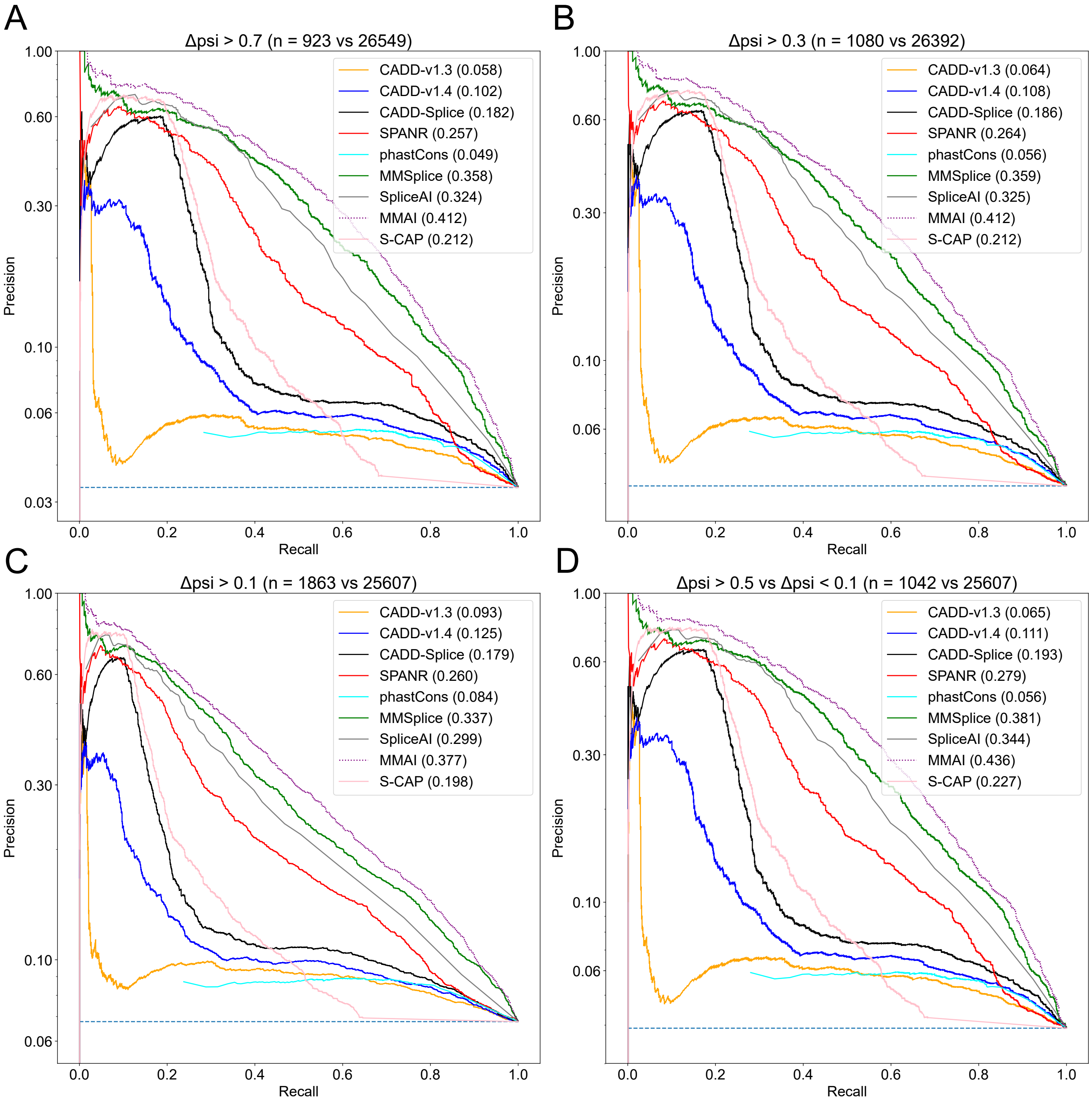

Fig S3: CADD v1.4 benefited from dbscSNV. Precision-Recall curves for different CADD versions, dbscSNV, MMSplice and SpliceAI. Panel (A) shows only the MFASS variants where the dbscSNV random forest score is defined. Panel (B) presents the remaining MFASS variants. While CADD v1.4 outperforms CADD v1.3 on variants with scores available from dbscSNV, the performance of both versions is very similar where dbscSNV is not defined.

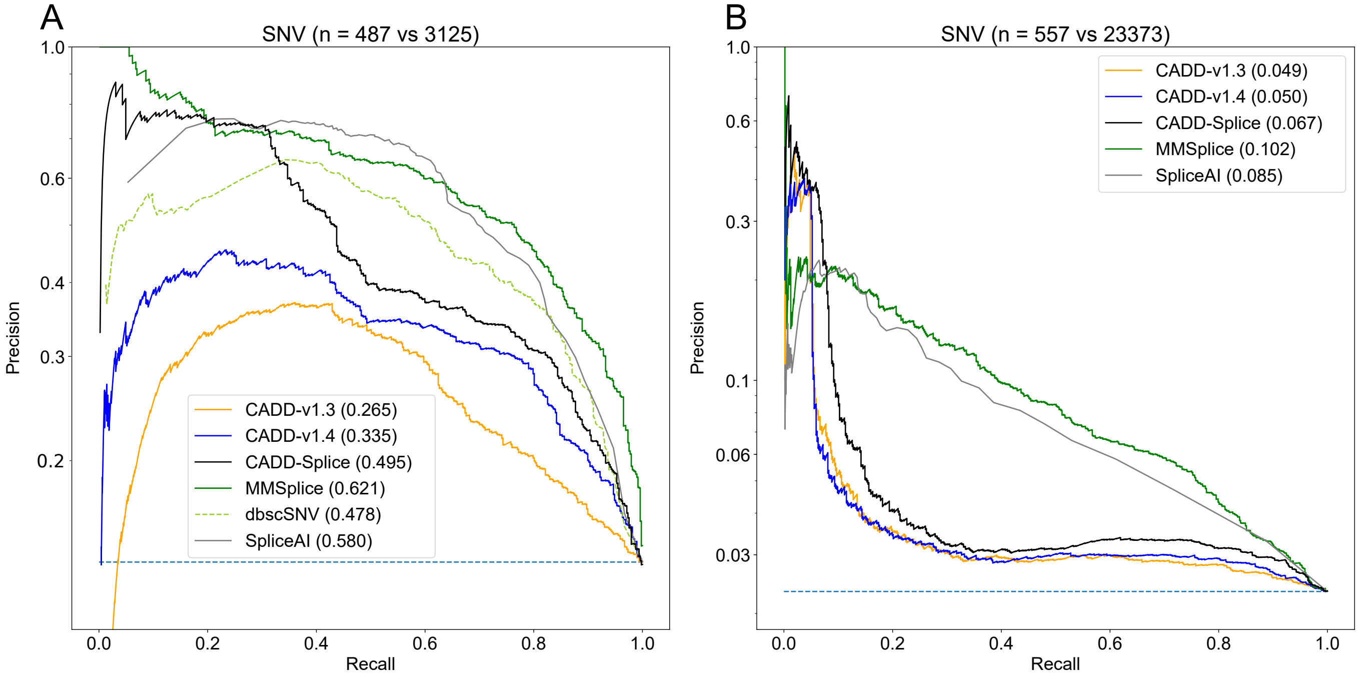

Fig S4: Separation of SpliceAI submodel scores in CADD training data set. Histograms of SpliceAI donor gain and acceptor loss submodel scores for 10 million SNV of the proxy-deleterious (simulated) and proxy-benign (human derived) category from the CADD training data set. Masked variants (loss variants that influence a known splice junction and gain variants outside of a known splice junction) are depleted in the simulated category for variants with high scores. Other variants (loss outside of known splice junction/gain at a known splice junction) are not depleted. Not all training set variants could be annotated by SpliceAI. Number of variants per plot is given as n = number simulated / number of human derived variants.

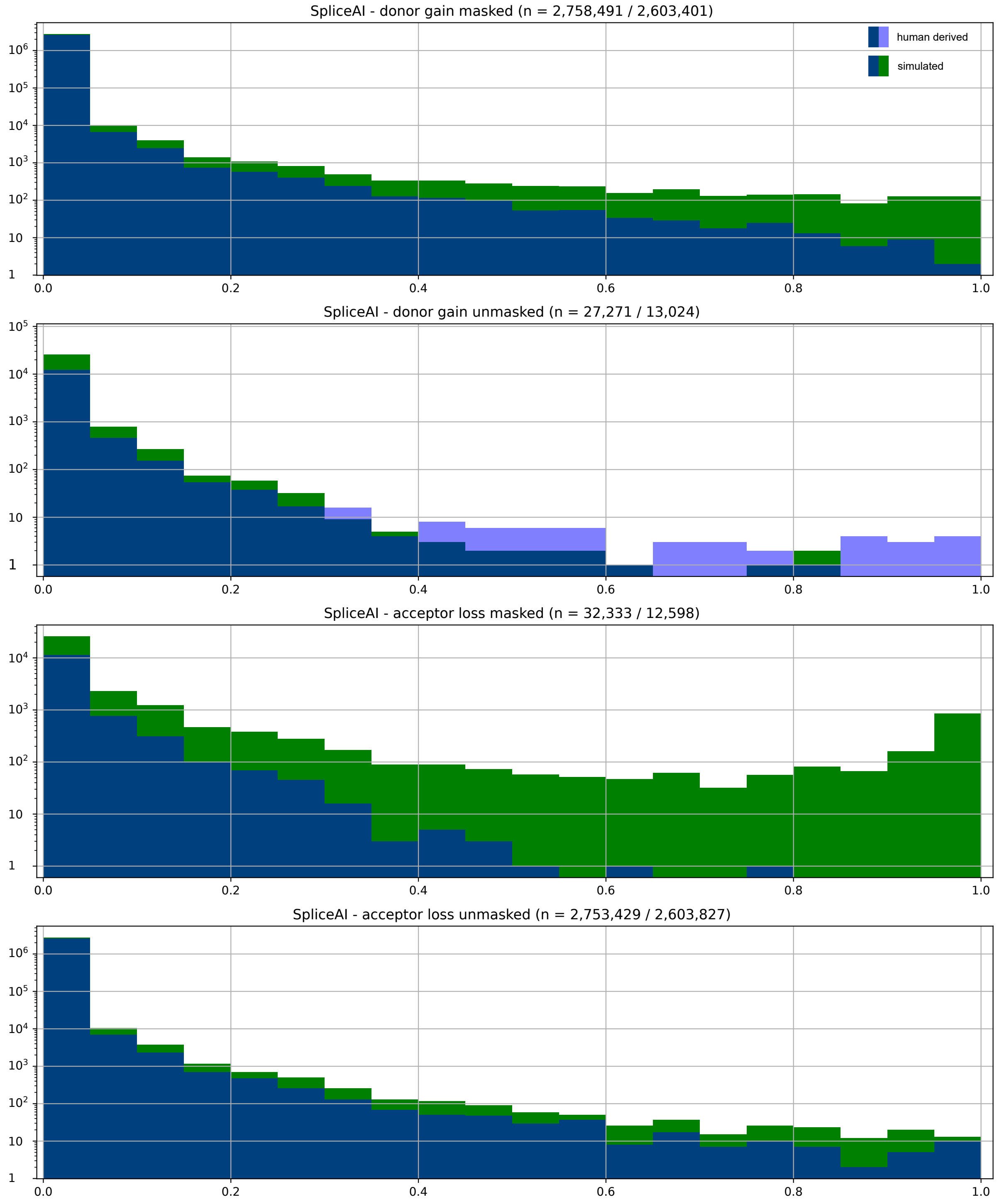

Fig S5: MMSplice submodel scores in CADD training data set. Histograms of MMSplice scores for SNV of the proxy-deleterious (simulated) and proxy-benign (human derived) category from the CADD training data set. Number of variants per plot is given as n = number of simulated variants / number of human derived variants. MMSplice submodel scores are the result of subtracting the prediction for reference allele from that of the sequence of the alternative allele. Functional changes are expected to result in different model predictions between the alleles and thus give a positive or negative score. For negative scores, a depletion of human derived variants is observed. This enrichment gets more pronounced for increasingly negative scores. For positive scores, a depletion of simulated variants is observed (most prominently for acceptor and donor model). This is in violation to the expectation of functional variants being depleted in the human derived relative to the simulated set in CADD training. It most likely results from the inversion of reference/alternative alleles necessary for scoring human derived variants, while relying on transcript models of the human reference.

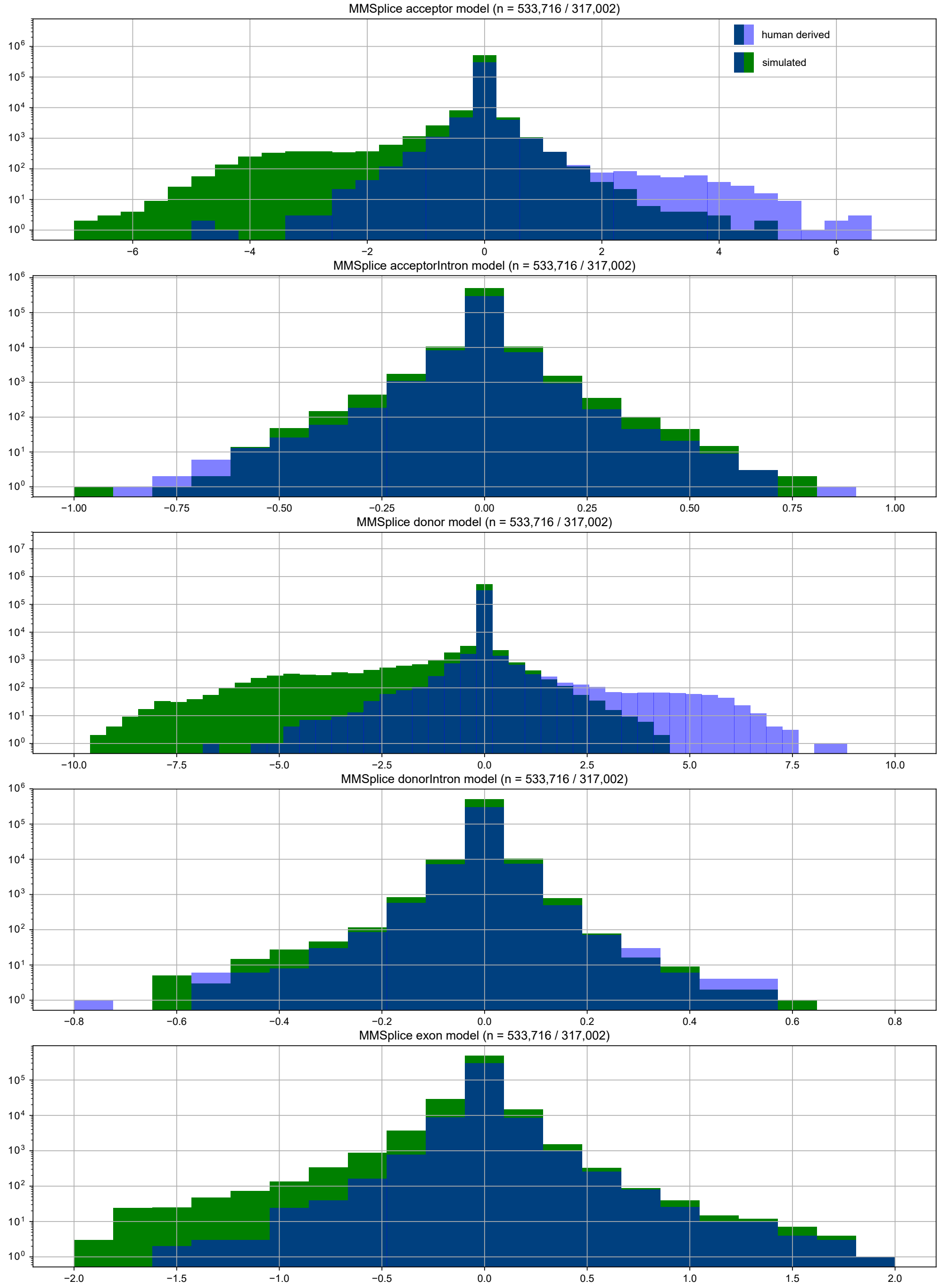

Fig S6: ClinVar pathogenic vs. common gnomAD variants for additional variant effect classes. CADD-Splice modestly outperforms previous versions of CADD in distinguishing between pathogenic variants from ClinVar and common population variants (MAF > 0.05) from gnomAD in terms of auROC. All three tested CADD versions are better than specialized scores when tested on all SNV (A) or missense SNV (B). For synonymous SNV (C), the CADD-Splice model performs similar to SpliceAI and better than previous versions. Only very few common variants are found at canonical splice sites (D). On this limited variant set, specialized splice scores perform worse than for other splicing related variant types.

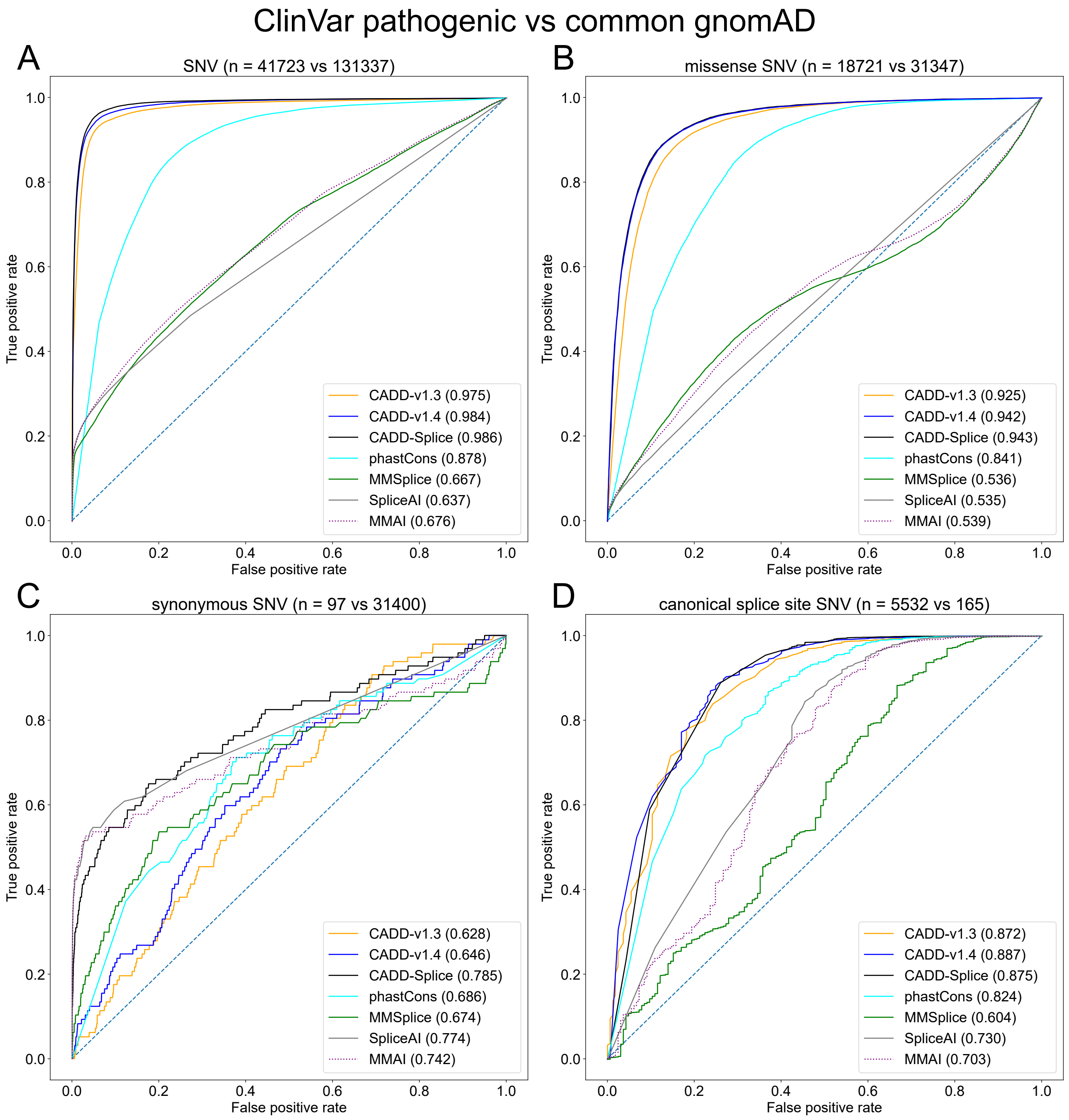

Fig S7: Depletion and enrichment of non-splicing gnomAD variants. Depletion and enrichment of variants grouped by frequency and CADD score bins, with CADD-Splice outperforming previous versions. At high CADD scores frequent (mean allele frequency MAF > 0.001) and rare (allele count > 1) variants are depleted and singletons (observed once in gnomAD) enriched. The most recent versions of CADD (v1.4 and CADD-Splice) enrich singleton variants and deplete frequent variants at higher relative scores with very similar ratios for synonymous (left), missense (middle) and intronic variants (right).

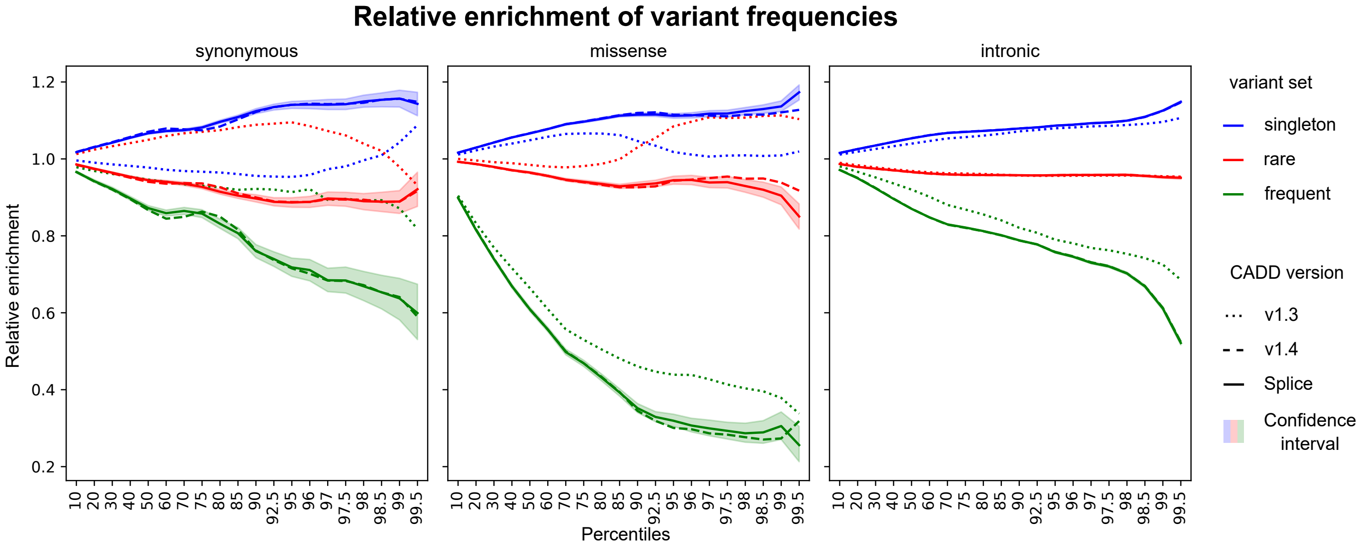

Fig S8: ClinVar likely-pathogenic vs. rare gnomAD variants. While ClinVar pathogenic variants are used in training of splicing classifiers, variants assigned the clinical significance "likely-pathogenic" may be used as a non-overlapping validation set. Similarly, common variants (MAF > 0.05) are often used as benign sets, but non-singleton rare variants (MAF < 0.05, allele count > 1) are also under purifying selection. Therefore, we use the comparison ClinVar likely-pathogenic vs. rare gnomAD variants as a replacement and additional validation set. All three tested CADD versions are better than specialized scores when tested on all SNV (A), intronic (C) or missense SNV (D). For splice site variants (B), the splice scores SpliceAI and MMSplice perform as good or better than previous versions of CADD, with CADD-Splice performing better than all. For synonymous SNV (E), the CADD-Splice model performs similar to SpliceAI and better than previous versions. On canonical splice site variants (F), specialized splice scores perform worse than for other splicing related variant types and do not improve performance of CADD-Splice in comparison to CADD GRCh37-v1.4.

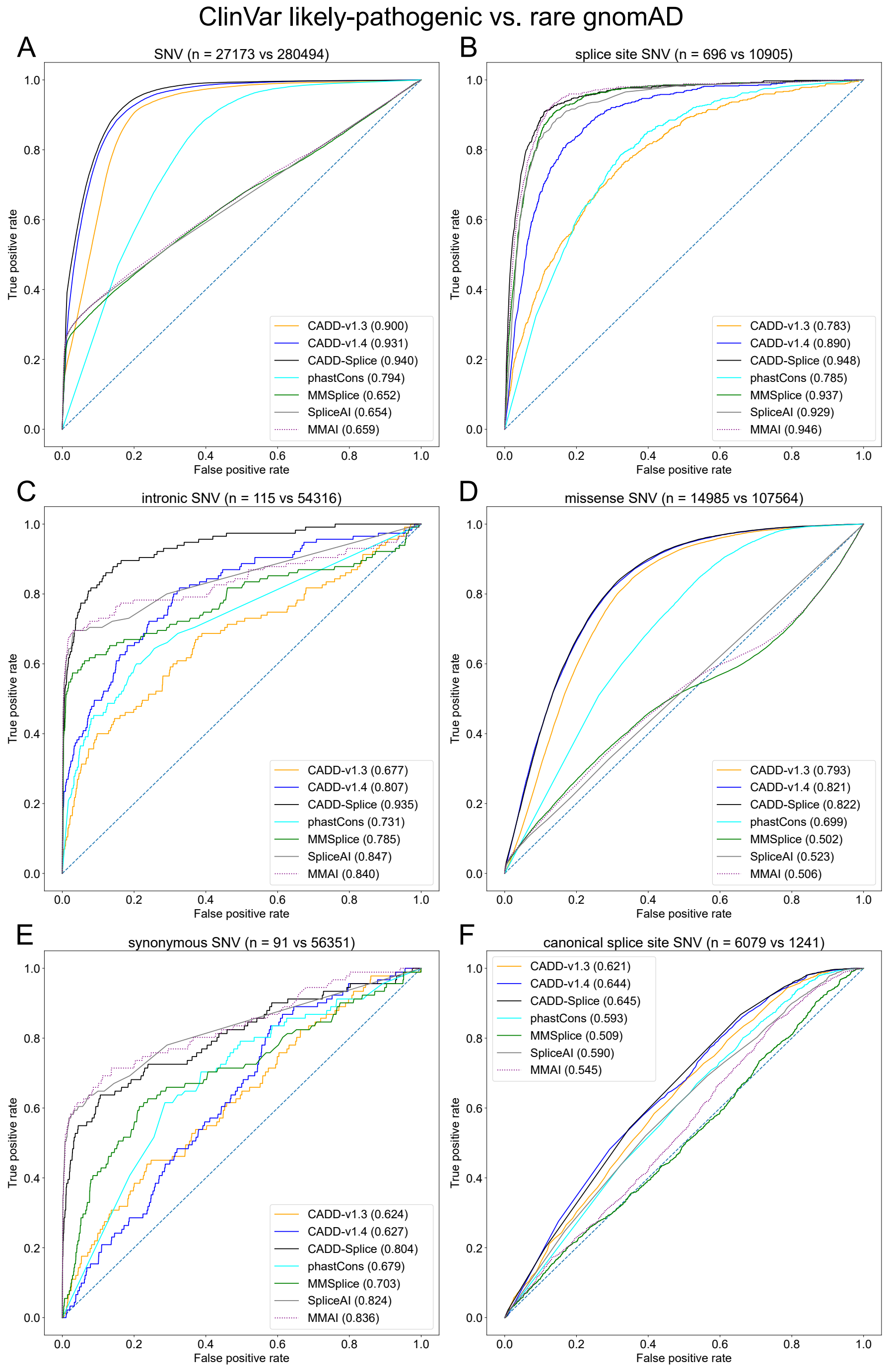

Fig S9: Comparing additional scores for ClinVar likely-pathogenic vs. rare gnomAD variants. Scores of additional tools are compared on ClinVar likely-pathogenic SNV and rare population variants (MAF<0.05, allele count > 1) from gnomAD. As not all tools score all variants, either the number of variants compared depends on the chosen set of scores or performance of tools is impacted by how missing scores are imputed. Here, S-CAP is compared by first selecting only SNV that can be scored (A-D). Secondly, SNV with missing S-CAP scores are imputed as benign (score of 0) in panels (E-H). While coverage of canonical splice site SNV (B & F) is very high and of similar frequency in likely-pathogenic and proxy neutral variants, other categories such as intronic are missing S-CAP scores for many variants. Across panels B-D and F-H, S-CAP misses 5,980 out of 66,608 (9%) variants.

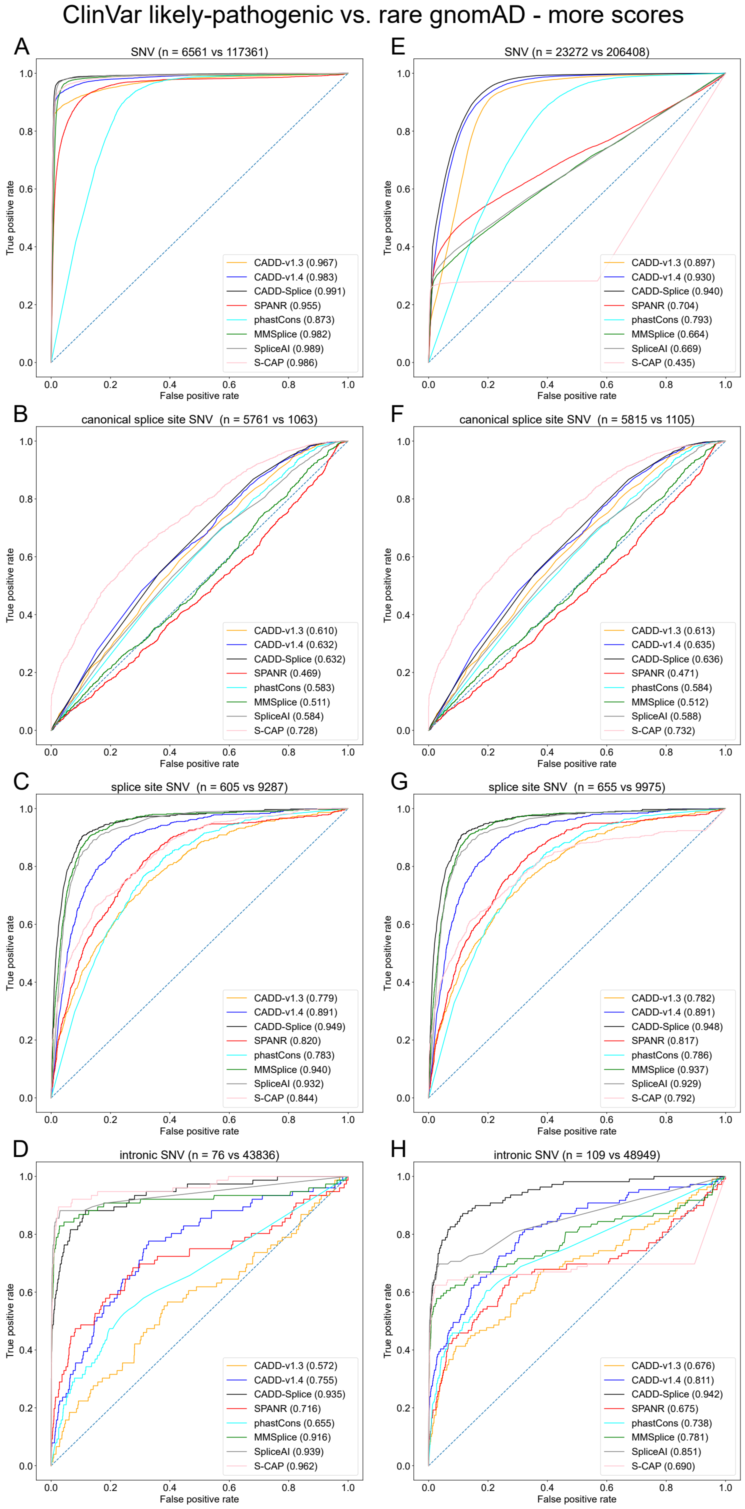

Fig S10: CADD GRCh38 - ClinVar pathogenic vs. common gnomAD. Using the same parameters as for CADD-Splice, we trained a CADD model for GRCh38 that includes MMSplice and SpliceAI. As for GRCh37, performance on all SNV classes (A) and splice site variants (B) for separating pathogenic variants from ClinVar from common variants in gnomAD (MAF > 0.05) improves with v1.6 over the previous release v1.5. In order to compare performance across genome builds, variants were lifted from GRCh37 to GRCh38 post scoring.

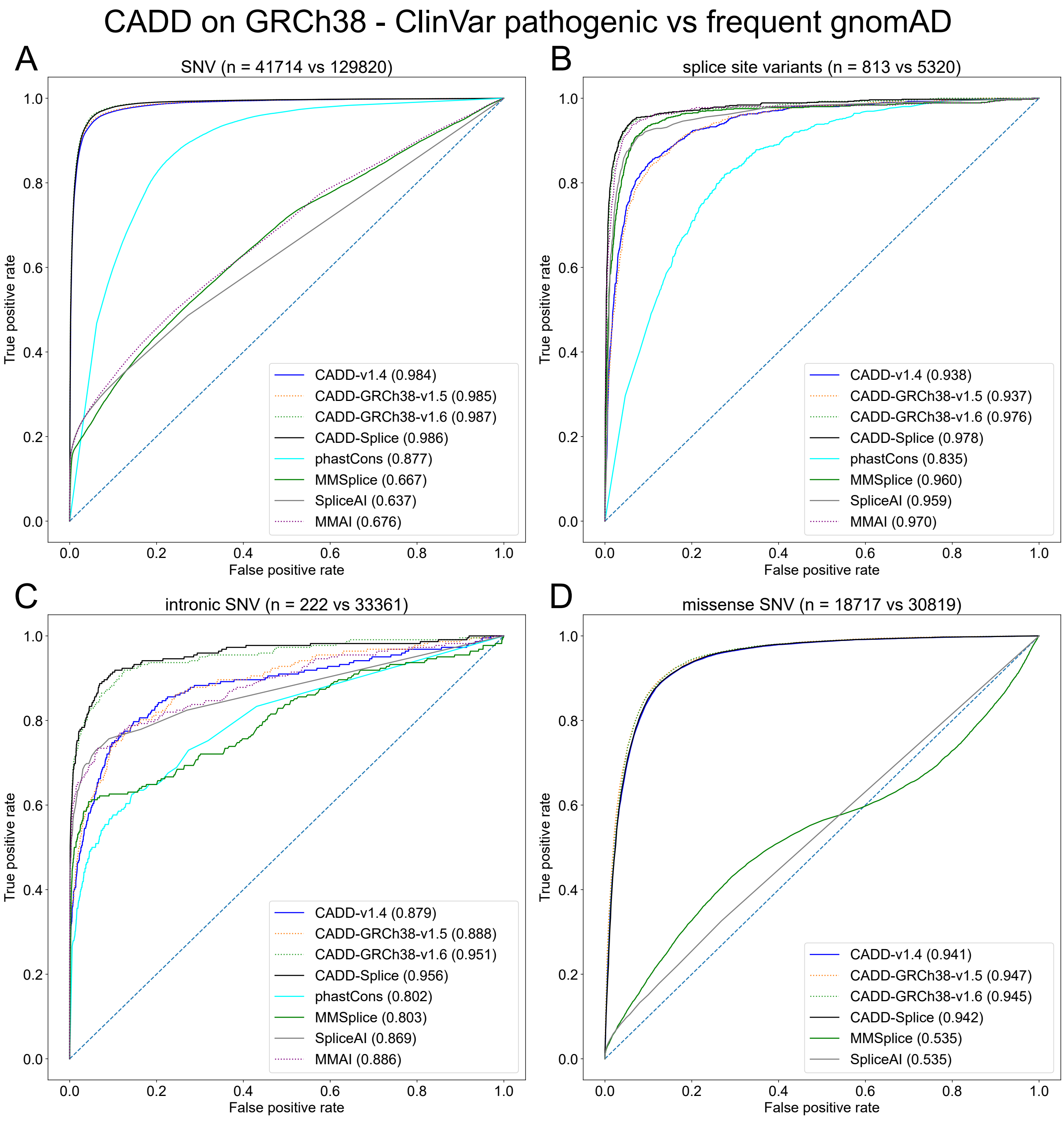

Supplemental Tables

Table S1: CADD-Splice (CADD GRCh37-v1.6) annotations. Columns in CADD v1.6 (GRCh37 model) output files including underlying annotations. Name fields in parentheses describe annotations that are not used for calculating the CADD score and only serve for the user to interpret the prediction results.

	Name	Type	Description
1	(Chrom)	string	Chromosome
2	(Pos)	int	Position (1-based)
3	Ref	factor	Reference allele (default: N)
4	Alt	factor	Observed allele (default: N)
5	Type	factor	Event type (SNV, DEL, INS)
6	Length	int	Number of inserted/deleted bases
7	(Annotype)	factor	CodingTranscript, Intergenic, MotifFeature, NonCodingTranscript, RegulatoryFeature, Transcript
8	Consequence	factor	VEP consequence, priority selected by potential impact (default: INTERGENIC)
9	(ConsScore)	int	Custom deleterious score assigned to Consequence
10	(ConsDetail)	string	Trimmed VEP consequence prior to simplification
11	GC	float	Percent GC in a window of +/- 75bp (default: 0.42)
12	CpG	float	Percent CpG in a window of +/- 75bp (default: 0.02)
13	MotifECount	int	Total number of overlapping motifs (default: 0)
14	(MotifEName)	string	Name of sequence motif the position overlaps
15	MotifEHIPos	bool	Is the position considered highly informative for an overlapping motif by VEP (default: 0)
16	MotifEScoreChng	float	VEP score change for the overlapping motif site (default: 0)
17	oAA	factor	Reference amino acid (default: unknown)
18	nAA	factor	Amino acid of observed variant (default: unknown)
19	(GeneID)	string	ENSEMBL GeneID
20	(FeatureID)	string	ENSEMBL feature ID (Transcript ID or regulatory feature ID)
21	(GeneName)	string	GeneName provided in ENSEMBL annotation
22	(CCDS)	string	Consensus Coding Sequence ID
23	(Intron)	string	Intron number/Total number of exons
24	(Exon)	string	Exon number/Total number of exons
25	cDNApos	float	Base position from transcription start (default: 0*)
26	relcDNApos	float	Relative position in transcript (default: 0)
27	CDSpos	float	Base position from coding start (default: 0*)
28	relCDSpos	float	Relative position in coding sequence (default: 0)
29	protPos	float	Amino acid position from coding start (default: 0*)
30	relprotPos	float	Relative position in protein codon (default: 0)
31	Domain	factor	Domain annotation inferred from VEP annotation (ncoils, sigp, lcompl, hmmpanther, ndomain = "other named domain") (default: UD)
32	Dst2Splice	float	Distance to splice site in 20bp; positive: exonic, negative: intronic (default: 0)
33	Dst2SplType	factor	Closest splice site is ACCEPTOR or DONOR (default: unknown)
34	MinDistTSS	float	Distance to closest Transcribed Sequence Start (TSS) (default: 5.5)
35	MinDistTSE	float	Distance to closest Transcribed Sequence End (TSE) (default: 5.5)
36	SIFTcat	factor	SIFT category of change (default: UD)

37	SIFTval	float	SIFT score (default: 0*)
38	PolyPhenCat	factor	PolyPhen category of change (default: UD)
39	PolyPhenVal	float	PolyPhen score (default: 0*)
40	priPhCons	float	Primate PhastCons conservation score (excl. human) (default: 0.115)
41	mamPhCons	float	Mammalian PhastCons conservation score (excl. human) (default: 0.079)
42	verPhCons	float	Vertebrate PhastCons conservation score (excl. human) (default: 0.094)
43	priPhyloP	float	Primate PhyloP score (excl. human) (default: -0.033)
44	mamPhyloP	float	Mammalian PhyloP score (excl. human) (default: -0.038)
45	verPhyloP	float	Vertebrate PhyloP score (excl. human) (default: 0.017)
46	bStatistic	int	Background selection score (default: 800)
47	targetScan	int	targetscan (default: 0*)
48	mirSVR-Score	float	mirSVR-Score (default: 0*)
49	mirSVR-E	float	mirSVR-E (default: 0)
50	mirSVR-Aln	int	mirSVR-Aln (default: 0)
51	cHmmTssA	float	Proportion of 127 cell types in cHmmTssA state (default: 0.0667*)
52	cHmmTssAFlnk	float	Proportion of 127 cell types in cHmmTssAFlnk state (default: 0.0667)
53	cHmmTxFlnk	float	Proportion of 127 cell types in cHmmTxFlnk state (default: 0.0667)
54	cHmmTx	float	Proportion of 127 cell types in cHmmTx state (default: 0.0667)
55	cHmmTxWk	float	Proportion of 127 cell types in cHmmTxWk state (default: 0.0667)
56	cHmmEnhG	float	Proportion of 127 cell types in cHmmEnhG state (default: 0.0667)
57	cHmmEnh	float	Proportion of 127 cell types in cHmmEnh state (default: 0.0667)
58	cHmmZnfRpts	float	Proportion of 127 cell types in cHmmZnfRpts state (default: 0.0667)
59	cHmmHet	float	Proportion of 127 cell types in cHmmHet state (default: 0.0667)
60	cHmmTssBiv	float	Proportion of 127 cell types in cHmmTssBiv state (default: 0.0667)
61	cHmmBivFlnk	float	Proportion of 127 cell types in cHmmBivFlnk state (default: 0.0667)
62	cHmmEnhBiv	float	Proportion of 127 cell types in cHmmEnhBiv state (default: 0.0667)
63	cHmmReprPC	float	Proportion of 127 cell types in cHmmReprPC state (default: 0.0667)
64	cHmmReprPCWk	float	Proportion of 127 cell types in cHmmReprPCWk state (default: 0.0667)
65	cHmmQuies	float	Proportion of 127 cell types in cHmmQuies state (default: 0.0667)
66	GerpRS	float	Gerp element score (default: 0)
67	GerpRSpval	float	Gerp element p-Value (default: 0)
68	GerpN	float	Neutral evolution score defined by GERP++ (default: 1.91)
69	GerpS	float	Rejected Substitution score defined by GERP++ (default: -0.2)
70	TFBS	float	Number of different overlapping ChIP transcription factor binding sites (default: 0)
71	TFBSPeaks	float	Number of overlapping ChIP transcription factor binding site peaks summed over different cell types/tissue (default: 0)
72	TFBSPeaksMax	float	Maximum value of overlapping ChIP transcription factor binding site peaks across cell types/tissue (default: 0)
73	tOverlapMotifs	float	Number of overlapping predicted TF motifs (default: 0)
74	motifDist	float	Reference minus alternate allele difference in nucleotide frequency within an predicted overlapping motif (default: 0)
75	Segway	factor	Result of genomic segmentation algorithm (default: unknown)
76	EncH3K27Ac	float	Maximum ENCODE H3K27 acetylation level (default: 0)
77	EncH3K4Me1	float	Maximum ENCODE H3K4 methylation level (default: 0)
78	EncH3K4Me3	float	Maximum ENCODE H3K4 trimethylation level (default: 0)
79	EncExp	float	Maximum ENCODE expression value (default: 0)

80	EncNucleo	float	Maximum of ENCODE Nucleosome position track score (default: 0)
81	EncOCC	int	ENCODE open chromatin code (default: 5)
82	EncOCCombPVal	float	ENCODE combined p-Value (PHRED-scale) of Faire, Dnase, polII, CTCF, Myc evidence for open chromatin (default: 0)
83	EncOCDNasePVal	float	p-Value (PHRED-scale) of Dnase evidence for open chromatin (default: 0)
84	EncOCFairePVal	float	p-Value (PHRED-scale) of Faire evidence for open chromatin (default: 0)
85	EncOCpolIIPVal	float	p-Value (PHRED-scale) of polII evidence for open chromatin (default: 0)
86	EncOCctcfPVal	float	p-Value (PHRED-scale) of CTCF evidence for open chromatin (default: 0)
87	EncOCmycPVal	float	p-Value (PHRED-scale) of Myc evidence for open chromatin (default: 0)
88	EncOCDNaseSig	float	Peak signal for Dnase evidence of open chromatin (default: 0)
89	EncOCFaireSig	float	Peak signal for Faire evidence of open chromatin (default: 0)
90	EncOCpolIISig	float	Peak signal for polII evidence of open chromatin (default: 0)
91	EncOCctcfSig	float	Peak signal for CTCF evidence of open chromatin (default: 0)
92	EncOCmycSig	float	Peak signal for Myc evidence of open chromatin (default: 0)
93	Grantham	float	Grantham score: oAA,nAA (default: 0*)
94	SpliceAI-acc-gain	float	Masked SpliceAI acceptor gain score (default: 0*)
95	SpliceAI-acc-loss	float	Masked SpliceAI acceptor loss score (default: 0)
96	SpliceAI-don-gain	float	Masked SpliceAI donor gain score (default: 0)
97	SpliceAI-don-loss	float	Masked SpliceAI donor loss score (default: 0)
98	MMSp_acceptorIntron	float	MMSplice acceptor intron (intron 3’) score (default: 0)
99	MMSp_acceptor	float	MMSplice acceptor score (default: 0)
100	MMSp_exon	float	MMSplice exon score (default: 0)
101	MMSp_donor	float	MMSplice donor score (default: 0)
102	MMSp_donorIntron	float	MMSplice donor intron (intron 5’) score (default: 0)
103	Dist2Mutation	float	Distance between the closest BRAVO SNV up and downstream (position itself excluded) (default: 0*)
104	Freq100bp	int	Number of frequent (MAF > 0.05) gnomAD SNV in 100 bp window nearby (default: 0)
105	Rare100bp	int	Number of rare (MAF < 0.05) gnomAD SNV in 100 bp window nearby (default: 0)
106	Sngl100bp	int	Number of single occurrence gnomAD SNV in 100 bp window nearby (default: 0)
107	Freq1000bp	int	Number of frequent (MAF > 0.05) gnomAD SNV in 1000 bp window nearby (default: 0)
108	Rare1000bp	int	Number of rare (MAF < 0.05) gnomAD SNV in 1000 bp window nearby (default: 0)
109	Sngl1000bp	int	Number of single occurrence gnomAD SNV in 1000 bp window nearby (default: 0)
110	Freq10000bp	int	Number of frequent (MAF > 0.05) gnomAD SNV in 10000 bp window nearby (default: 0)
111	Rare10000bp	int	Number of rare (MAF < 0.05) gnomAD SNV in 10000 bp window nearby (default: 0)
112	Sngl10000bp	int	Number of single occurrence gnomAD SNV in 10000 bp window nearby (default: 0)
113	dbscSNV-ada_score	float	Adaboost classifier score from dbscSNV (default: 0*)
114	dbscSNV-rf_score	float	Random forest classifier score from dbscSNV (default: 0*)
115	(RawScore)	float	Raw score from the model

116	(PHRED)	float	CADD PHRED Score

* A Boolean indicator variable was created in order to handle undefined values. Note that often indicators represent more than one annotation. They are created for only (the first) one if the covered genomic regions are identical.

Table S2: CADD GRCh38-v1.6 annotations. Columns in CADD v1.6 (GRCh38 model) output files including underlying annotations. Name fields in parentheses describe annotations that are not used for calculating the CADD score and only serve for the user to interpret the prediction results.

	Name	Type	Description
1	(Chrom)	string	Chromosome
2	(Pos)	int	Position (1-based)
3	Ref	factor	Reference allele (default: N)
4	Alt	factor	Observed allele (default: N)
5	Type	factor	Event type (SNV, DEL, INS)
6	Length	int	Number of inserted/deleted bases
7	(AnnoType)	factor	CodingTranscript, Intergenic, MotifFeature, NonCodingTranscript, RegulatoryFeature, Transcript
8	Consequence	factor	VEP consequence, priority selected by potential impact (default: INTERGENIC)
9	(ConsScore)	int	Custom deleterious score assigned to Consequence
10	(ConsDetail)	string	Trimmed VEP consequence prior to simplification
11	GC	float	Percent GC in a window of +/- 75bp (default: 0.42)
12	CpG	float	Percent CpG in a window of +/- 75bp (default: 0.02)
13	motifECount	int	Total number of overlapping motifs (default: 0)
14	(motifEName)	string	Name of sequence motif the position overlaps
15	motifEHIPos	bool	Is the position considered highly informative for an overlapping motif by VEP (default: 0)
16	motifEScoreChng	float	VEP score change for the overlapping motif site (default: 0)
17	oAA	factor	Reference amino acid (default: unknown)
18	nAA	factor	Amino acid of observed variant (default: unknown)
19	(GeneID)	string	ENSEMBL GeneID
20	(FeatureID)	string	ENSEMBL feature ID (Transcript ID or regulatory feature ID)
21	(GeneName)	string	GeneName provided in ENSEMBL annotation
22	(CCDS)	string	Consensus Coding Sequence ID
23	(Intron)	string	Intron number/Total number of exons
24	(Exon)	string	Exon number/Total number of exons
25	cDNApos	float	Base position from transcription start (default: 0*)
26	relcDNApos	float	Relative position in transcript (default: 0)
27	CDSpos	float	Base position from coding start (default: 0*)
28	relCDSpos	float	Relative position in coding sequence (default: 0)
29	protPos	float	Amino acid position from coding start (default: 0*)
30	relProtPos	float	Relative position in protein codon (default: 0)
31	Domain	factor	Domain annotation inferred from VEP annotation (ncoils, sigp, lcompl, hmmpanther, ndomain = "other named domain") (default: UD)
32	Dst2Splice	float	Distance to splice site in 20bp; positive: exonic, negative: intronic (default: 0)
33	Dst2SplType	factor	Closest splice site is ACCEPTOR or DONOR (default: unknown)
34	minDistTSS	float	Distance to closest Transcribed Sequence Start (TSS) (default: 5.5)
35	minDistTSE	float	Distance to closest Transcribed Sequence End (TSE) (default: 5.5)
36	SIFTcat	factor	SIFT category of change (default: UD)
37	SIFTval	float	SIFT score (default: 0*)
38	PolyPhenCat	factor	PolyPhen category of change (default: UD)

39	PolyPhenVal	float	PolyPhen score (default: 0*)
40	priPhCons	float	Primate PhastCons conservation score (excl. human) (default: 0.0)
41	mamPhCons	float	Mammalian PhastCons conservation score (excl. human) (default: 0.0)
42	verPhCons	float	Vertebrate PhastCons conservation score (excl. human) (default: 0.0)
43	priPhyloP	float	Primate PhyloP score (excl. human) (default: -0.029)
44	mamPhyloP	float	Mammalian PhyloP score (excl. human) (default: -0.005)
45	verPhyloP	float	Vertebrate PhyloP score (excl. human) (default: 0.042)
46	bStatistic	int	Background selection score (default: 800)
47	targetScan	int	targetscan (default: 0*)
48	mirSVR-Score	float	mirSVR-Score (default: 0*)
49	mirSVR-E	float	mirSVR-E (default: 0)
50	mirSVR-Aln	int	mirSVR-Aln (default: 0)
51	cHmm_E1	float	Number of 48 cell types in chromHMM state E1_poised (default: 1.92*)
52	cHmm_E2	float	Number of 48 cell types in chromHMM state E2_repressed (default: 1.92)
53	cHmm_E3	float	Number of 48 cell types in chromHMM state E3_dead (default: 1.92)
54	cHmm_E4	float	Number of 48 cell types in chromHMM state E4_dead (default: 1.92)
55	cHmm_E5	float	Number of 48 cell types in chromHMM state E5_repressed (default: 1.92)
56	cHmm_E6	float	Number of 48 cell types in chromHMM state E6_repressed (default: 1.92)
57	cHmm_E7	float	Number of 48 cell types in chromHMM state E7_weak (default: 1.92)
58	cHmm_E8	float	Number of 48 cell types in chromHMM state E8_gene (default: 1.92)
59	cHmm_E9	float	Number of 48 cell types in chromHMM state E9_gene (default: 1.92)
60	cHmm_E10	float	Number of 48 cell types in chromHMM state E10_gene (default: 1.92)
61	cHmm_E11	float	Number of 48 cell types in chromHMM state E11_gene (default: 1.92)
62	cHmm_E12	float	Number of 48 cell types in chromHMM state E12_distal (default: 1.92)
63	cHmm_E13	float	Number of 48 cell types in chromHMM state E13_distal (default: 1.92)
64	cHmm_E14	float	Number of 48 cell types in chromHMM state E14_distal (default: 1.92)
65	cHmm_E15	float	Number of 48 cell types in chromHMM state E15_weak (default: 1.92)
66	cHmm_E16	float	Number of 48 cell types in chromHMM state E16_tss (default: 1.92)
67	cHmm_E17	float	Number of 48 cell types in chromHMM state E17_proximal (default: 1.92)
68	cHmm_E18	float	Number of 48 cell types in chromHMM state E18_proximal (default: 1.92)
69	cHmm_E19	float	Number of 48 cell types in chromHMM state E19_tss (default: 1.92)
70	cHmm_E20	float	Number of 48 cell types in chromHMM state E20_poised (default: 1.92)
71	cHmm_E21	float	Number of 48 cell types in chromHMM state E21_dead (default: 1.92)
72	cHmm_E22	float	Number of 48 cell types in chromHMM state E22_repressed (default: 1.92)
73	cHmm_E23	float	Number of 48 cell types in chromHMM state E23_weak (default: 1.92)
74	cHmm_E24	float	Number of 48 cell types in chromHMM state E24_distal (default: 1.92)
75	cHmm_E25	float	Number of 48 cell types in chromHMM state E25_distal (default: 1.92)
76	GerpRS	float	Gerp element score (default: 0)
77	GerpRSpval	float	Gerp element p-Value (default: 0)
78	GerpN	float	Neutral evolution score defined by GERP++ (default: 3.0)
79	GerpS	float	Rejected Substitution score defined by GERP++ (default: -0.2)

80	tOverlapMotifs	float	Number of overlapping predicted TF motifs
81	motifDist	float	Reference minus alternate allele difference in nucleotide frequency within an predicted overlapping motif (default: 0)
82	EncodeH3K4me1-sum	float	Sum of Encode H3K4me1 levels (from 13 cell lines) (default: 0.76)
83	EncodeH3K4me1-max	float	Maximum Encode H3K4me1 level (from 13 cell lines) (default: 0.37)
84	EncodeH3K4me2-sum	float	Sum of Encode H3K4me2 levels (from 14 cell lines) (default: 0.73)
85	EncodeH3K4me2-max	float	Maximum Encode H3K4me2 level (from 14 cell lines) (default: 0.37)
86	EncodeH3K4me3-sum	float	Sum of Encode H3K4me3 levels (from 14 cell lines) (default: 0.81)
87	EncodeH3K4me3-max	float	Maximum Encode H3K4me3 level (from 14 cell lines) (default: 0.38)
88	EncodeH3K9ac-sum	float	Sum of Encode H3K9ac levels (from 13 cell lines) (default: 0.82)
89	EncodeH3K9ac-max	float	Maximum Encode H3K9ac level (from 13 cell lines) (default: 0.41)
90	EncodeH3K9me3-sum	float	Sum of Encode H3K9me3 levels (from 14 cell lines) (default: 0.81)
91	EncodeH3K9me3-max	float	Maximum Encode H3K9me3 level (from 14 cell lines) (default: 0.38)
92	EncodeH3K27ac-sum	float	Sum of Encode H3K27ac levels (from 14 cell lines) (default: 0.74)
93	EncodeH3K27ac-max	float	Maximum Encode H3K27ac level (from 14 cell lines) (default: 0.36)
94	EncodeH3K27me3-sum	float	Sum of Encode H3K27me3 levels (from 14 cell lines) (default: 0.93)
95	EncodeH3K27me3-max	float	Maximum Encode H3K27me3 level (from 14 cell lines) (default: 0.47)
96	EncodeH3K36me3-sum	float	Sum of Encode H3K36me3 levels (from 10 cell lines) (default: 0.71)
97	EncodeH3K36me3-max	float	Maximum Encode H3K36me3 level (from 10 cell lines) (default: 0.39)
98	EncodeH3K79me2-sum	float	Sum of Encode H3K79me2 levels (from 13 cell lines) (default: 0.64)
99	EncodeH3K79me2-max	float	Maximum Encode H3K79me2 level (from 13 cell lines) (default: 0.34)
100	EncodeH4K20me1-sum	float	Sum of Encode H4K20me1 levels (from 11 cell lines) (default: 0.88)
101	EncodeH4K20me1-max	float	Maximum Encode H4K20me1 level (from 11 cell lines) (default: 0.47)
102	EncodeH2AFZ-sum	float	Sum of Encode H2AFZ levels (from 13 cell lines) (default: 0.9)
103	EncodeH2AFZ-max	float	Maximum Encode H2AFZ level (from 13 cell lines) (default: 0.42)
104	EncodeDNase-sum	float	Sum of Encode DNase-seq levels (from 12 cell lines) (default: 0.0)
105	EncodeDNase-max	float	Maximum Encode DNase-seq level (from 12 cell lines) (default: 0.0)
106	EncodetotalRNA-sum	float	Sum of Encode totalRNA-seq levels (from 10 cell lines always minus and plus strand) (default: 0.0)
107	EncodetotalRNA-max	float	Maximum Encode totalRNA-seq level (from 10 cell lines, minus and plus strand separately) (default: 0.0)
108	Grantham	float	Grantham score: oAA,nAA (default: 0*)
109	SpliceAI-acc-gain	float	Masked SpliceAI acceptor gain score (default: 0*)
110	SpliceAI-acc-loss	float	Masked SpliceAI acceptor loss score (default: 0)
111	SpliceAI-don-gain	float	Masked SpliceAI donor gain score (default: 0)
112	SpliceAI-don-loss	float	Masked SpliceAI donor loss score (default: 0)
113	MMSp_acceptorIntron	float	MMSplice acceptor intron (intron 3’) score (default: 0)
114	MMSp_acceptor	float	MMSplice acceptor score (default: 0)
115	MMSp_exon	float	MMSplice exon score (default: 0)
116	MMSp_donor	float	MMSplice donor score (default: 0)
117	MMSp_donorIntron	float	MMSplice donor intron (intron 5’) score (default: 0)
118	Dist2Mutation	float	Distance between the closest BRAVO SNV up and downstream (position itself excluded) (default: 0*)
119	Freq100bp	int	Number of frequent (MAF > 0.05) BRAVO SNV in 100 bp window nearby (default: 0)
120	Rare100bp	int	Number of rare (MAF < 0.05) BRAVO SNV in 100 bp window nearby (default: 0)

121	Sngl100bp	int	Number of single occurrence BRAVO SNV in 100 bp window nearby (default: 0)
122	Freq1000bp	int	Number of frequent (MAF > 0.05) BRAVO SNV in 1000 bp window nearby (default: 0)
123	Rare1000bp	int	Number of rare (MAF < 0.05) BRAVO SNV in 1000 bp window nearby (default: 0)
124	Sngl1000bp	int	Number of single occurrence BRAVO SNV in 1000 bp window nearby (default: 0)
125	Freq10000bp	int	Number of frequent (MAF > 0.05) BRAVO SNV in 10000 bp window nearby (default: 0)
126	Rare10000bp	int	Number of rare (MAF < 0.05) BRAVO SNV in 10000 bp window nearby (default: 0)
127	Sngl10000bp	int	Number of single occurrence BRAVO SNV in 10000 bp window nearby (default: 0)
128	EnsembleRegulatory-Feature	factor	Matches in the Ensemble Regulatory Built (similar to annotype) (default: NA)
129	dbscSNV-ada_score	float	Adaboost classifier score from dbscSNV (default: 0*)
130	dbscSNV-rf_score	float	Random forest classifier score from dbscSNV (default: 0*)
131	RemapOverlapTF	int	Remap number of different transcription factors binding (default: -0.5)
132	RemapOverlapCL	int	Remap number of different transcription factor - cell line combinations binding (default: -0.5)
133	(RawScore)	float	Raw score from the model
134	(PHRED)	float	CADD PHRED Score

* A Boolean indicator variable was created in order to handle undefined values. Note that often indicators represent more than one annotation. They are created for only (the first) one if the covered genomic regions are identical.

Table S3: Data set used in CADD-Splice development. Developing CADD-Splice, we used various data sets, many of which are the default for training and evaluating CADD models as previously described [1, 2]. The MFASS data [3] was primarily used to identify splice predictors and to reevaluate performance of CADD models.

Data set	Source	Used for
MFASS	https://krishna.gs.washington.edu/download/CADD-development/v1.6/validation/MFASS/, [3]	feature exploration, AUC comparisons
Human derived	https://krishna.gs.washington.edu/download/CADD-development/v1.6/training_data/GRCh37/, [1]	feature selection, positive† CADD training labels
Simulated variants	https://krishna.gs.washington.edu/download/CADD-development/v1.6/training_data/GRCh37/, [1]	feature selection, negative† CADD training labels
ClinVar - pathogenic	https://krishna.gs.washington.edu/download/CADD-development/v1.6/validation/clinVar/, [4]	negative† set in AUC comparisons, model validation and test *
ClinVar - likely pathogenic	https://krishna.gs.washington.edu/download/CADD-development/v1.6/validation/clinVar/, [4]	negative† set in AUC comparisons, model test
gnomAD exome , MAF > 0.05	https://krishna.gs.washington.edu/download/CADD-development/v1.6/validation/gnomad/, [5]	positive† set in AUC comparisons, model validation and test *
gnomAD exome, MAF < 0.05	https://krishna.gs.washington.edu/download/CADD-development/v1.6/validation/gnomad/, [5]	positive† set in AUC comparisons, model test
DMS data set of BRCA1, YAP1, DLG4, TPMT	https://krishna.gs.washington.edu/download/CADD-development/v1.6/validation/DMS/, [6]	Score correlation, model validation
Enhancer & promoter MPRA	https://krishna.gs.washington.edu/download/CADD-development/v1.6/validation/MPRA/, [7–9]	Score correlation, model validation
TP53 frequencies	https://krishna.gs.washington.edu/download/CADD-development/v1.6/validation/tp53/, [10]	Score correlation, model validation
HBB severity classes	https://krishna.gs.washington.edu/download/CADD-	Class-wise score separation (Kruskal-Wallis test), model validation

	development/v1.6/validation/HBB/, [11]	

† negative corresponds to suspected pathogenic variants while positive corresponds to suspected neutral
* chromosome hold-out split between validation and test

References

1. Kircher M, Witten DM, Jain P, et al (2014) A general framework for estimating the relative pathogenicity of human genetic variants. Nat Genet 46:310–315. https://doi.org/10.1038/ng.2892
2. Rentzsch P, Witten D, Cooper GM, et al (2019) CADD: predicting the deleteriousness of variants throughout the human genome. Nucleic Acids Res 47:D886–D894. https://doi.org/10.1093/nar/gky1016
3. Cheung R, Insigne KD, Yao D, et al (2019) A Multiplexed Assay for Exon Recognition Reveals that an Unappreciated Fraction of Rare Genetic Variants Cause Large-Effect Splicing Disruptions. Mol Cell 73:183–194. https://doi.org/10.1016/j.molcel.2018.10.037
4. Landrum MJ, Lee JM, Benson M, et al (2018) ClinVar: improving access to variant interpretations and supporting evidence. Nucleic Acids Res 46:D1062–D1067. https://doi.org/10.1093/nar/gkx1153
5. Karczewski KJ, Francioli LC, Tiao G, et al (2020) The mutational constraint spectrum quantified from variation in 141,456 humans. Nature 581:434–443. https://doi.org/10.1038/s41586-020-2308-7
6. Gray VE, Hause RJ, Luebeck J, et al (2018) Quantitative Missense Variant Effect Prediction Using Large-Scale Mutagenesis Data. Cell Syst 6:116-124.e3. https://doi.org/10.1016/j.cels.2017.11.003
7. Patwardhan RP, Lee C, Litvin O, et al (2009) High-resolution analysis of DNA regulatory elements by synthetic saturation mutagenesis. Nat Biotechnol 27:1173–1175. https://doi.org/10.1038/nbt.1589
8. Patwardhan RP, Hiatt JB, Witten DM, et al (2012) Massively parallel functional dissection of mammalian enhancers *in vivo*. Nat Biotechnol 30:265–270. https://doi.org/10.1038/nbt.2136
9. Kircher M, Xiong C, Martin B, et al (2019) Saturation mutagenesis of twenty disease-associated regulatory elements at single base-pair resolution. Nat Commun 10:3583. https://doi.org/10.1038/s41467-019-11526-w
10. Bouaoun L, Sonkin D, Ardin M, et al (2016) TP53 Variations in Human Cancers: New Lessons from the IARC TP53 Database and Genomics Data. Hum Mutat 37:865–876. https://doi.org/10.1002/humu.23035
11. Giardine B, Baal S van, Kaimakis P, et al (2007) HbVar database of human hemoglobin variants and thalassemia mutations: 2007 update. Hum Mutat 28:206–206. https://doi.org/10.1002/humu.9479
